# *CXCL8* producing macrophages shape gastric cancer outcomes

**DOI:** 10.1007/s12672-026-05452-9

**Published:** 2026-06-17

**Authors:** Lin Zhang, Hanming Yu, Han Zhang, Yang Zhou, Zhanjun Miao, Yuanzeng Zhu

**Affiliations:** 1 Comprehensive Surgical Ward, Henan Provincial People’s Hospital, Weiwu Road, Zhengzhou, People’s Republic of China; 2 Department of Pulmonary and Critical Care Medicine, The Fourth Affiliated Hospital of China Medical University, No. 77 Puhe Road, Shenyang, China; 3 Department of General Surgery, Henan Provincial People’s Hospital, Weiwu Road, Zhengzhou, People’s Republic of China; 4 Department of Hepatobiliary and Gastrointestinal Surgery, Hongxing Hospital, 13th Division, Xinjiang Production and Construction Corps, Hami, Xinjiang People’s Republic of China

**Keywords:** Stomach neoplasms, Chemokines, Macrophages, Prognostic, Interleukin-8

## Abstract

**Background:**

Gastric cancer (GC) remains a major cause of cancer-related mortality worldwide, highlighting the need for reliable prognostic biomarkers. Although chemokines critically regulate the tumor microenvironment (TME), their integrated prognostic value and mechanistic roles in GC remain unclear. We aimed to develop a chemokine-based prognostic signature and elucidate its underlying cellular basis.

**Methods:**

Transcriptomic data from TCGA and GEO cohorts were integrated to construct and validate a prognostic model using least absolute shrinkage and selection operator (LASSO) Cox regression based on six differentially expressed chemokines. Associations between the risk score and TME characteristics, tumor mutation burden (TMB), and drug sensitivity were evaluated. Single-cell RNA sequencing (scRNA-seq) was performed to determine the cellular origin and functional role of the key chemokine *CXCL8*.

**Results:**

A six-chemokine signature stratified patients into high- and low-risk groups with significantly different overall survival (OS; *p* = 0.003) and progression-free survival (PFS; *p* < 0.001). The risk score was an independent prognostic factor, and the corresponding nomogram demonstrated good predictive performance (C-index = 0.680). The high-risk group exhibited elevated TMB and reduced chemosensitivity. scRNA-seq analysis identified macrophages as the principal source of *CXCL8*. These *CXCL8*-high macrophages displayed a pro-tumorigenic phenotype and functioned as key communication hubs within the TME, particularly through *SPP1* signaling.

**Conclusion:**

This chemokine-based signature provides clinically relevant prognostic stratification in GC and implicates the *CXCL8*–macrophage axis as a potential therapeutic target.

**Supplementary Information:**

The online version contains supplementary material available at 10.1007/s12672-026-05452-9.

## Introduction

Gastric cancer (GC) ranks as the fifth most common malignancy worldwide, with an age-standardized incidence of 9.2 per 100,000 individuals in 2022, and represents the fourth leading cause of cancer-related mortality, with a death rate of 6.1 per 100,000 [1]. Globally, more than 1 million new cases were diagnosed in 2020, resulting in approximately 769,000 deaths [2]. The highest prevalence has been documented in Eastern Asia, Europe’s eastern regions, and Latin American countries [3]. Although the global incidence has declined over recent decades, mortality remains substantial, with 5-year survival rates consistently below 30% [4, 5]. Despite advances in surgical techniques, radiotherapy, chemotherapy, and targeted therapies [6, 7], most patients are diagnosed at intermediate or advanced stages, contributing to persistently poor clinical outcomes [8, 9]. Therefore, the development of more precise molecular classification systems and robust prognostic models is urgently needed to guide personalized therapeutic strategies and improve patient survival [10].

Chemokines are a family of small secreted cytokines that regulate immune cell trafficking and intercellular communication. They play fundamental roles in physiological immune surveillance [11, 12] and in shaping the tumor microenvironment (TME) [13, 14]. Emerging evidence indicates that specific chemokines, such as *CXCL10*, modulate immune cell infiltration patterns [15], whereas *CXCL6* promotes angiogenesis through endothelial cell activation [16, 17]. Other chemokines have been implicated in tumor metastasis and immune evasion [18]. Collectively, these findings underscore the pivotal role of chemokines in tumor initiation and progression. However, the global expression landscape, immunomodulatory functions, and prognostic value of chemokines in GC remain insufficiently defined and warrant systematic investigation [19].

In this study, we integrated The Cancer Genome Atlas (TCGA) and transcriptomic datasets to systematically identify chemokines with prognostic relevance in GC. A risk model based on chemokine expression profiles was constructed to predict patient outcomes. The CIBERSORT algorithm was applied to evaluate differences in immune cell infiltration and to explore associations between the risk score and the tumor immune microenvironment. The predictive performance of the chemokine-based risk model developed in the TCGA cohort was externally validated using the GSE84473 and GSE183904 datasets. These findings provide a theoretical basis for elucidating immunoregulatory mechanisms in GC and for advancing individualized therapeutic strategies.

## Materials and methods

### Data collection and preprocessing

The data used in this study were obtained from The TCGA and the Gene Expression Omnibus (GEO) databases. Gene expression data, clinical information, and mutation data for GC samples were downloaded from the TCGA database. Additionally, the gastric cancer datasets GSE84473 and GSE183904 from the GEO database were also utilized.

Data processing includes standardization of gene expression data, log2 transformation, and gene ID transformation. All data will be processed uniformly to ensure their quality and comparability.

### Construction of a chemokine-based prognostic risk signature

Differential expression analysis between GC and adjacent normal tissues was performed using the “limma” R package, with thresholds of *p* < 0.05 and |log2 fold change (logFC)| ≥ 1. Chemokine-related genes were extracted based on official gene symbols, and prognostic relevance was evaluated using univariate Cox proportional hazards regression analysis implemented in the “survival” R package. Genes that were both differentially expressed and significantly associated with overall survival were defined as differentially expressed prognostic chemokines (DEPCs).

To reduce overfitting and enhance model robustness, LASSO Cox regression analysis was conducted using the “glmnet” R package. The optimal penalty parameter (λ) was determined through 10-fold cross-validation.

A risk score for each patient was calculated as follows:

$$ \begin{gathered} {\text{Risk score}} \hfill \\ = \Sigma \left( {{\text{Expression level of gene}} \times {\text{corresponding LASSO coefficient}}} \right) \hfill \\ \end{gathered} $$

Patients were stratified into high- and low-risk groups according to the median risk score in the training cohort.

### Validation and evaluation of the prognostic risk model

The prognostic value of the risk score was assessed using univariate and multivariate Cox proportional hazards regression analyses to determine its independence from conventional clinicopathological variables. Hazard ratios (HRs) and 95% confidence intervals (CIs) were visualized using forest plots.

Kaplan–Meier survival curves were generated using the “survminer” R package, and differences in overall survival (OS) and progression-free survival (PFS) between high- and low-risk groups were evaluated using the log-rank test. Time-dependent receiver operating characteristic (ROC) curves were constructed using the “timeROC” R package to assess the predictive performance of the risk model at 1-, 3-, and 5-year time points, and the corresponding area under the curve (AUC) values were calculated.

A prognostic nomogram integrating independent clinical variables (age, T stage) and the risk score was developed based on multivariate Cox regression analysis and stepwise model selection, and visualized using the “regplot” package. The calibration performance of the nomogram was evaluated using calibration plots, and its clinical utility was further assessed by decision curve analysis (DCA).

The optimal cutoff value for risk stratification was determined using the “surv_cutpoint” function implemented in the “survminer” package, allowing classification of patients into high- and low-risk groups.

### Enrichment analysis

Differentially expressed genes (DEGs) between the high- and low-risk groups were identified using the “limma” R package with thresholds of |log2 fold change (logFC)| > 1 and adjusted *P* < 0.05.

Functional enrichment analysis was performed using the “clusterProfiler” R package, including Gene Ontology (GO) and Kyoto Encyclopedia of Genes and Genomes (KEGG) pathway analyses. Enriched terms were considered statistically significant at adjusted *P* < 0.05.

Gene Set Enrichment Analysis (GSEA) was conducted to further explore biological differences between risk groups. The “c2.cp.kegg.v2023.1.Hs.symbols.gmt” and “h.all.v2023.1.Hs.symbols.gmt” gene sets were obtained from the Molecular Signatures Database (MSigDB). Pathways with nominal *P* < 0.05 and false discovery rate (FDR) q < 0.25 were considered significantly enriched. Enrichment results were ranked according to normalized enrichment scores (NES).

In addition, gene set variation analysis (GSVA) was performed using the GSVA R package (version 1.46.0) to quantify pathway activity at the individual sample level.

### Analysis of somatic mutations and copy number variations

TCGA data enabled analysis of mutation frequencies in gastric cancer samples. The “maftools” package created waterfall plots depicting mutation frequencies across risk groups. Differences in mutation rates of key genes (*TP53*, *MUC16*, *TTN*, etc.) between risk groups were compared. To investigate associations between tumor mutation burden (TMB) and prognosis, patients were stratified into high-TMB and low-TMB cohorts. Kaplan-Meier survival curves analyzed survival differences between TMB groups. The combined influence of TMB and risk score on survival outcomes was also examined.

### Immune cell infiltration

Differences in immune cell infiltration between groups were assessed using the CIBERSORT algorithm, which analyzes expression data to characterize cellular composition of complex tissues from gene expression profiles [20]. We applied the LM22 leukocyte gene signature matrix (22 immune cell types), which is publicly available through the CIBERSORT portal at: https://cibersort.stanford.edu/.

### Analysis of public bulk RNA-seq data and scRNA-seq data

The bulk RNA-seq dataset GSE84437, comprising 483 gastric cancer patients, was obtained from the Gene Expression Omnibus (GEO). Differential expression analysis between tumor and normal gastric tissues was performed using the limma R package. Genes with an absolute log2 fold change (|logFC| > 0.5) and a false discovery rate (FDR < 0.05) were defined as differentially expressed chemokine-related genes and used for subsequent survival and clinical correlation analyses.

Single cell RNA sequencing dataset gse183904 was obtained from 、GEO, including tumor samples (*n* = 29) and adjacent normal samples (*n* = 11) from gastric cancer patients. After initial data loading, a total of 127,107 tumor derived cells and 31,342 normal derived cells were retained for downstream analysis after quality control.

Quality control filtering was performed using Seurat R package (version 4.3.0). Cells were retained if they met the following criteria: nFeature_RNA between 200 and 6,000, mitochondrial gene percentage ≤ 20%, and nCount_RNA ≤ 60,000. Genes detected in less than three cells were excluded.

After filtering, sctransform was used for data normalization and variance stabilization. Dimensionality reduction was performed by principal component analysis (PCA), and the harmony algorithm with default parameters was used to correct batch effects between samples. Clusters were visualized using uniform manifold approximation and projection (umap).

### Functional enrichment and pseudotime analysis

To investigate functional differences among cell subtypes, we performed gene set enrichment analysis using the clusterProfiler package (version 4.7.1003). This included KEGG pathway enrichment analysis using the KEGG gene sets, which are publicly available at: https://www.genome.jp/kegg/. In addition, single-sample gene set enrichment analysis (ssGSEA) was performed using gene sets from the Molecular Signatures Database (MSigDB, v7.6, https://www.gsea-msigdb.org/gsea/msigdb/). Enrichment results were visualized using the GseaVis package (version 0.0.8).

To explore macrophage developmental dynamics, macrophage subsets were isolated and analyzed using pseudotime analysis with the DDRTree algorithm in the Monocle 3 package (version 1.4.25). This approach allowed the reconstruction of pseudotime trajectories, providing insight into the continuous progression of macrophage differentiation.

### Cellular communication and metabolic profiling

To assess intercellular communication between macrophages and other cell types, we utilized the CellChat package (version 1.6.1), following its standard pipeline with default parameters. This approach allowed for the construction of a comprehensive cell-cell communication network, revealing the interactions and signaling pathways between different cell populations. Additionally, we inferred metabolic pathway activities to gain insight into the metabolic state of macrophages in the tissue microenvironment.

### Drug sensitivity analysis

Chemotherapy response profiles of TCGA-STAD patients were examined using pharmacogenomic data from the Genomics of Drug Sensitivity in Cancer (GDSC, release 2023, https://www.cancerrxgene.org/) database. The half-maximal inhibitory concentration (IC50) values for each drug were predicted using the “oncoPredict” R package (version 1.8.0, https://bioconductor.org/packages/oncoPredict/). The analysis focused on eight drugs commonly used in gastric cancer neoadjuvant chemotherapy, and differences in predicted drug sensitivity were compared between patient risk categories.

### Cell culture

Human gastric cancer MKN45 cells (Procell, Cat. No. CL-0292) and human monocytic THP-1 cells (Procell, Cat. No. CL-0233) were cultured in RPMI-1640 medium supplemented with 10% fetal bovine serum and 1% penicillin–streptomycin at 37 °C in a humidified incubator with 5% CO₂. THP-1 cells were differentiated into macrophage-like cells by treatment with phorbol 12-myristate 13-acetate (PMA, 100 ng/mL) for 24 h, followed by replacement with fresh medium prior to subsequent experiments.

### Quantitative PCR, ELISA and CCK-8 assay

Total RNA was extracted using TRIzol reagent according to the manufacturer’s protocol and reverse-transcribed into complementary DNA (cDNA). Quantitative real-time PCR (qRT–PCR) was performed using SYBR Green Master Mix on a real-time PCR system. Relative *CXCL8* mRNA expression levels were calculated using the 2^−ΔΔCt method, with GAPDH serving as the internal reference gene. The primer sequences for CXCL8 were as follows: forward 5′-ACTGAGAGTGATTGAGAGTGGAC-3′ and reverse 5′-AACCCTCTGCACCCAGTTTTC-3′.

CXCL8 protein concentrations in cell culture supernatants were quantified using a human CXCL8 ELISA kit (KE00453, Procell) in accordance with the manufacturer’s instructions.

Cell viability was evaluated using the Cell Counting Kit-8 (CCK-8) assay, and absorbance was measured at 450 nm using a microplate reader. For chemotherapeutic sensitivity assays, gastric cancer cells were treated with oxaliplatin (L-OHP) or 5-fluorouracil (5-FU) under three experimental conditions: control, co-culture with macrophages, and co-culture combined with an anti-*CXCL8* neutralizing antibody. Cells were exposed to a range of drug concentrations for 48 h to generate dose–response curves, followed by CCK-8 assessment. The half-maximal inhibitory concentration (IC_50_) values were calculated based on the fitted dose–response curves.

All experiments were performed in triplicate, and data are presented as mean ± standard deviation (SD). Statistical significance was defined as *P* < 0.05.

### Western blot

MKN45 Cells were lysed in RIPA buffer containing protease inhibitors, and protein concentrations were determined using a BCA assay. Equal amounts of protein were separated by SDS–PAGE and transferred onto PVDF membranes. After blocking with 5% non-fat milk, membranes were incubated overnight at 4 °C with mouse anti–PD-L1 antibody (Proteintech, Cat. No. 66248-1-Ig; 1:1000). Following incubation with HRP-conjugated secondary antibody, signals were detected using enhanced chemiluminescence.

### Immunohistochemistry

Paraffin-embedded gastric cancer and adjacent normal tissues were sectioned at 4 μm, deparaffinized, and subjected to antigen retrieval in citrate buffer (pH 6.0). After blocking endogenous peroxidase, sections were incubated overnight at 4 °C with rabbit anti-CXCL8 antibody (Proteintech, Cat. No. 27095-1-AP; 1:200). Signals were visualized using DAB, followed by hematoxylin counterstaining. Images were captured at ×100 magnification (scale bar = 100 μm).

### Transwell migration and invasion assays

Cell migration and invasion were assessed using Transwell chambers (8-µm pore size). For migration assays, MKN45 cells were seeded into the upper chamber in serum-free medium. For invasion assays, the upper chambers were pre-coated with Matrigel. Medium containing 10% FBS was added to the lower chamber as a chemoattractant. After incubation, cells on the lower surface were fixed, stained with crystal violet, and counted under a light microscope at ×100 magnification (scale bar = 100 μm).

### Statistical analysis

All bioinformatics and statistical analyses were performed using R software (version 4.4.3). Survival analyses were conducted using the Kaplan–Meier method, and differences between groups were assessed using the log-rank test. Univariate and multivariate Cox proportional hazards regression models were applied to identify independent prognostic factors. Comparisons between two groups were performed using the Wilcoxon rank-sum test, while multiple group comparisons were conducted using one-way analysis of variance (ANOVA), as appropriate. A two-sided P value < 0.05 was considered statistically significant.

All in vitro experimental data are presented as the mean ± standard deviation (SD) from at least three independent experiments. Statistical analyses were performed using one-way ANOVA followed by Tukey’s post hoc test in GraphPad Prism software (version 10.1; GraphPad Software, San Diego, CA, USA). A two-tailed P value < 0.05 was considered statistically significant.

## Results

### Identification of prognostic chemokines with differential expression

**Fig. 1:**
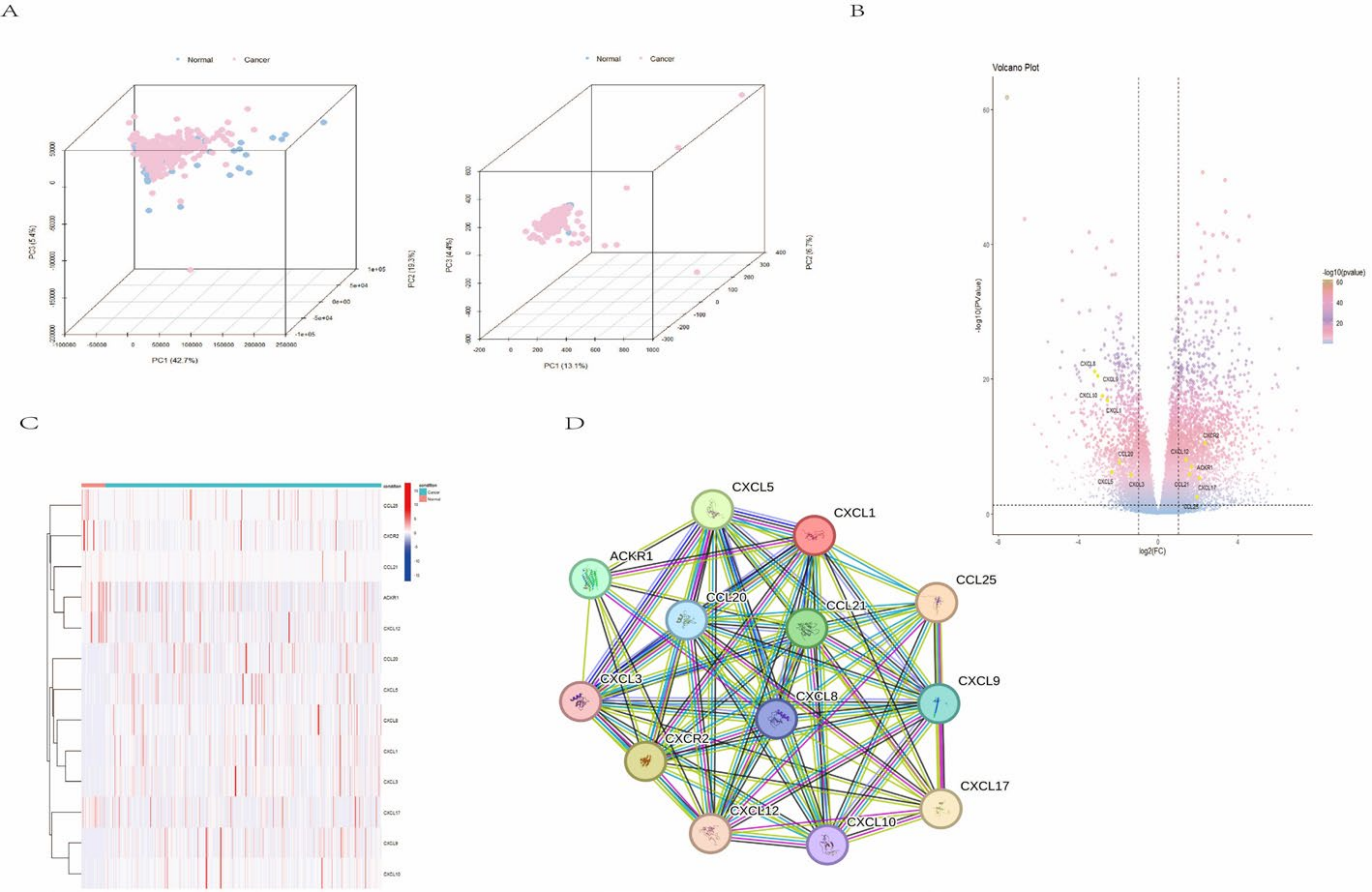
Identification of differentially expressed chemokines. **A** PCA plots before and after sample standardization. **B** Volcanic diagram of DEPC between tumor and normal. **C** Heat map of DEPCs. **D** The PPI network of DEPCs

PCA was first performed to standardize and reduce the dimensionality of the samples (Fig. 1A). Subsequently, the “limma” R package was used to identify 5947 DEGs between gastric cancer samples and normal gastric tissue, with p-values < 0.05 and absolute log2 fold changes > 1. Among these, 3216 genes were upregulated, and 2731 genes were downregulated in gastric cancer tissues. Figure 1B presents a volcano plot illustrating the distribution of these DEGs, with chemokine-related genes highlighted, including *CXCL1*, *CCL25*, *CXCR2*, *CXCL5*, *CCL21*, *ACKR1*, *CXCL9*, *CCL20*, *CXCL8*, *CXCL10*, *CXCL3*, *CXCL12*, and *CXCL17*, which were differentially expressed. Figure 1C shows a heatmap of the differential expression of these 13 chemokine genes between gastric cancer and normal gastric tissue samples. Figure 1D presents a protein-protein interaction (PPI) network constructed using the STRING database.

### Construction of a prognostic risk model based on chemokines

**Fig. 2:**
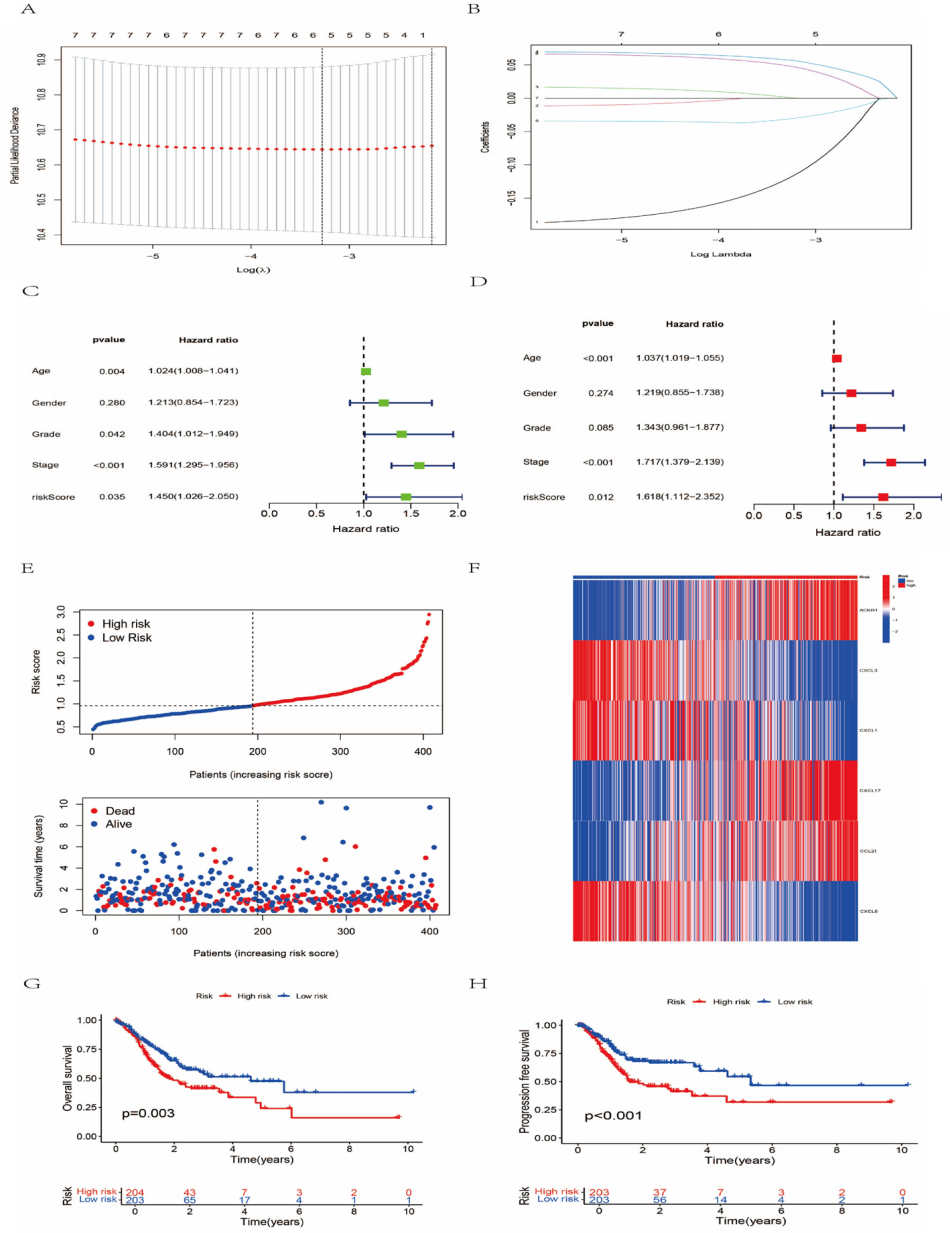
Evaluation of chemokine related prognostic models. The **A**, **B** cvfit and lambda trajectory diagrams illustrate the execution of LASSO regression following the minimum standard. **C** The results of univariate Cox regression evaluation of OS risk score and clinical pathological attributes in the TCGA cohort. **D** The results of multivariate Cox regression evaluation of OS risk score and clinical pathological attributes in the TCGA cohort. **E** Risk scores and corresponding overall survival outcomes in the TCGA cohort. **F** Heatmap of DEPCs in low-risk and high-risk groups. **G** The Kaplan Meier survival plot depicts the overall survival (OS) of the high-risk and low-risk groups in the TCGA cohort. **H** The Kaplan Meier survival plot depicts the PFS of high-risk and low-risk groups in the TCGA cohort

To develop a prognostic model, LASSO Cox regression analysis was performed using the expression profiles of DEPCs. Based on the optimal penalty parameter (λ), 9 chemokines were initially filtered, and 6 key genes—*ACKR1*, *CXCL8*, *CXCL1*, *CXCL3*, *CXCL17*, and *CCL21*—were selected for further analysis. Figure 2A depicts the expression trajectories of these genes as determined by LASSO regression, while Fig. 2B shows the confidence intervals for each λ value.

Univariate and multivariate Cox regression analyses were conducted to assess the prognostic independence of the risk score (Fig. 2C, D). Patients were stratified into high- and low-risk groups according to the optimal threshold for each cohort, with a higher mortality rate observed in the high-risk group (Fig. 2E). Figure 2F illustrates the differential expression of the six chemokine genes between the risk groups.

Kaplan–Meier survival analysis demonstrated significant differences between risk groups. High-risk patients exhibited shorter OS (Fig. 2G, *P* = 0.003) and PFS (Fig. 2H, *P* < 0.001).

### Construction of a nomogram and assessment of predictive performance

**Fig. 3:**
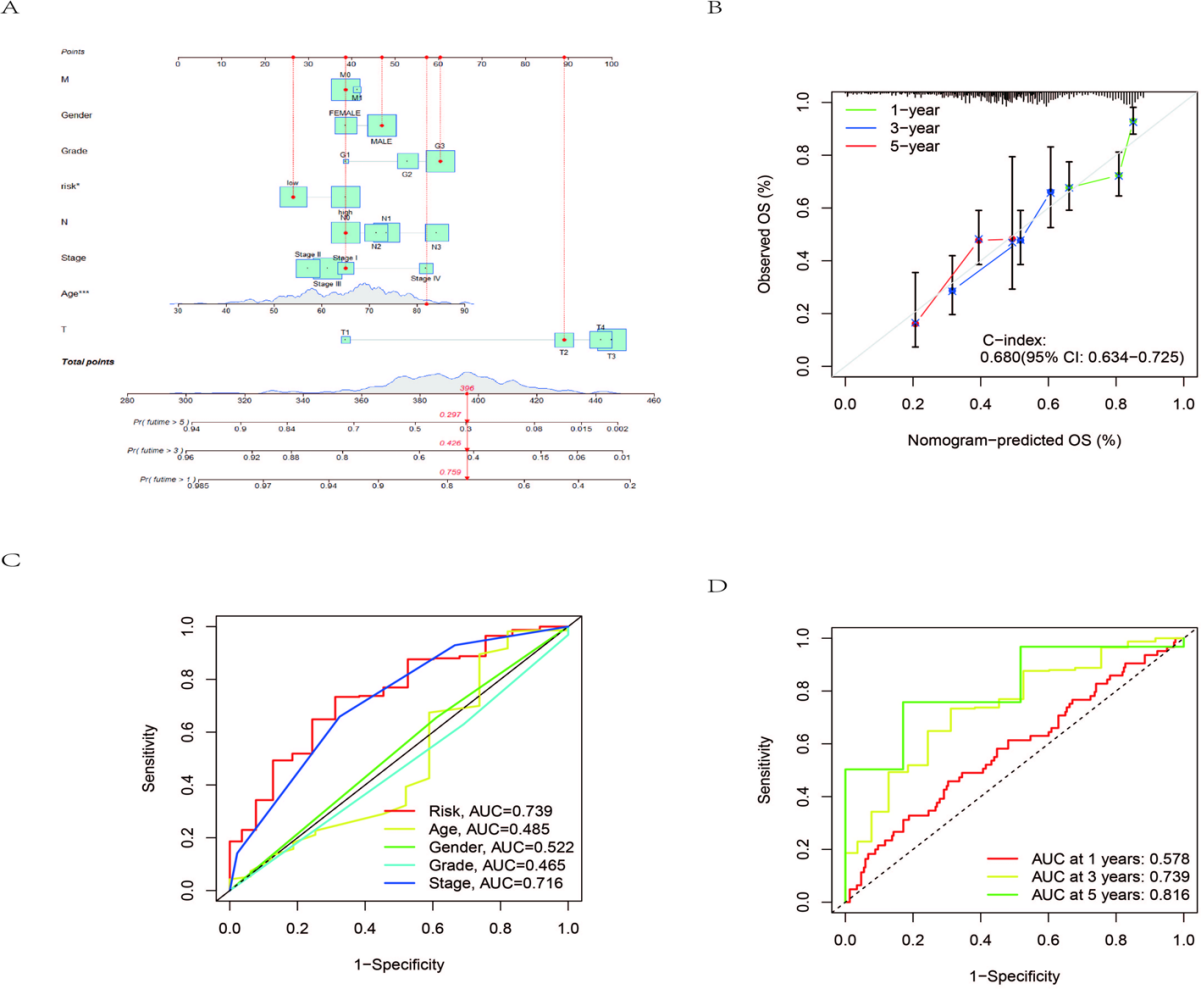
Evaluation of chemokine related prognostic models. **A** A column chart predicting the overall survival probabilities at 1, 3, and 5 years in the TCGA cohort. **B** Calibration chart of TCGA queue column chart. **C**, **D** The ROC analysis demonstrated the effectiveness of column charts in predicting 1-year, 3-year, and 5-year overall survival in the TCGA cohort

To enhance clinical applicability, a prognostic nomogram was constructed by integrating the chemokine-based risk score with clinicopathological variables (Fig. 3A). Age (*P* < 0.001) and the risk score (*P* < 0.05) were identified as significant contributors to the model. The nomogram converts individual predictors into a total point score, which is then used to estimate personalized 1-, 3-, and 5-year overall survival probabilities.

The model demonstrated good discriminative ability, with a concordance index (C-index) of 0.680 (95% CI: 0.634–0.725). Calibration curves showed good agreement between predicted and observed survival outcomes across all time points.

ROC curve analysis further evaluated the nomogram’s predictive performance. In the TCGA cohort, the AUC for the risk score (0.739) exceeded that of other traditional clinical indicators, indicating its independent prognostic value (Fig. 3C). The model’s AUC values for 1-, 3-, and 5-year overall survival were 0.578, 0.739, and 0.816, respectively, demonstrating improved predictive accuracy over time (Fig. 3D).

### Correlation between tumor microenvironment and risk model

**Fig. 4:**
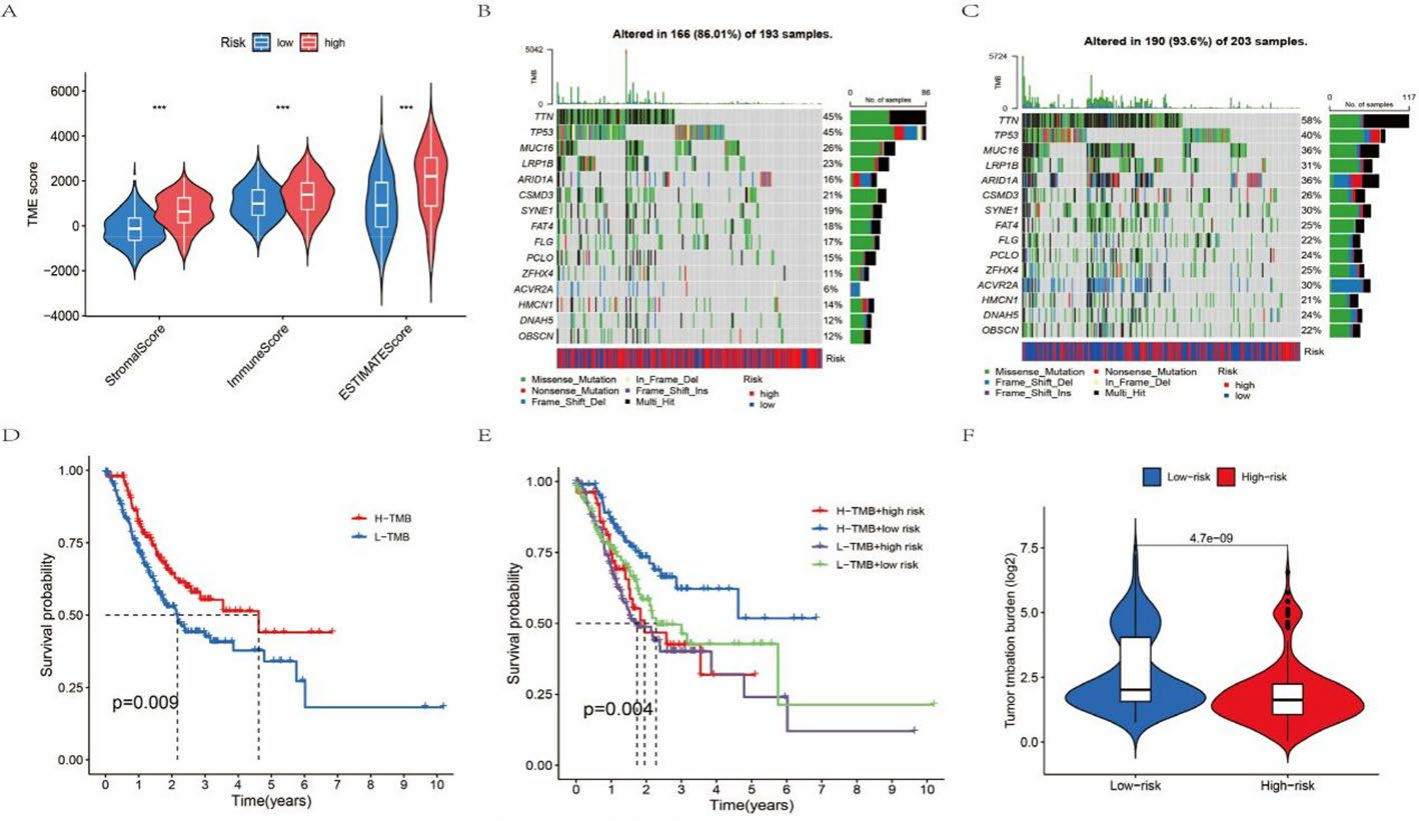
Tumor mutation burden. **A** The comparison of tumor microenvironment scores (StromalScore, ImmuneScore, ESTIMATESCore) among different risk groups showed that the high-risk group had higher levels of TME; **B**, **C** Waterfall plot of mutation frequency of the top 20 high-frequency mutated genes in low-risk group (**B**) and high-risk group (**C**) patients; **D** the overall survival rate of patients in the high TMB group was higher than that in the low TMB group (*p* = 0.009); **E** the survival analysis of the joint risk score and TMB grouping showed that the low-risk+high TMB group had the highest survival rate (*p* = 0.004); **F** the violin chart shows that the TMB of the high-risk group is significantly higher than that of the low-risk group (*p* < 0.0001)

To further explore the relationship between the risk score, TME, and TMB, differences in TME scores between high- and low-risk groups were assessed. The high-risk group exhibited significantly higher StromalScore, ImmuneScore, and ESTIMATEScore compared with the low-risk group (*P* < 0.001), suggesting increased immune cell infiltration and stromal components in high-risk patients (Fig. 4A).

Gene mutation frequencies in both risk groups were visualized using waterfall plots. Analysis of 193 samples revealed that genes such as *TTN*, *TP53*, and *MUC16* exhibited high mutation frequencies, with a greater mutation rate observed in the high-risk group (Fig. 4B). A similar trend was observed in 203 samples, where mutation frequencies of *TTN*, *TP53*, and *MUC16* remained high, and the high-risk group again displayed significantly elevated mutation rates relative to the low-risk group (Fig. 4C).

Survival analysis demonstrated that patients with high TMB (H-TMB) had significantly lower survival probabilities compared with those with low TMB (L-TMB) (*P* = 0.009), indicating an association between elevated TMB and poor prognosis (Fig. 4D). The combined analysis of TMB and risk groups showed that patients with both high-risk scores and high TMB had the lowest survival rates, whereas those with low-risk scores and low TMB had the most favorable prognosis, highlighting the synergistic effect of TMB and risk group on clinical outcomes (Fig. 4E).

Finally, a direct comparison of TMB between the two risk groups revealed that the high-risk group exhibited significantly higher TMB (*P* = 4.7e-09), suggesting a greater mutational burden that may contribute to adverse prognosis (Fig. 4F).

### Single-cell atlas reveals macrophages as the primary source of *CXCL8*

**Fig. 5:**
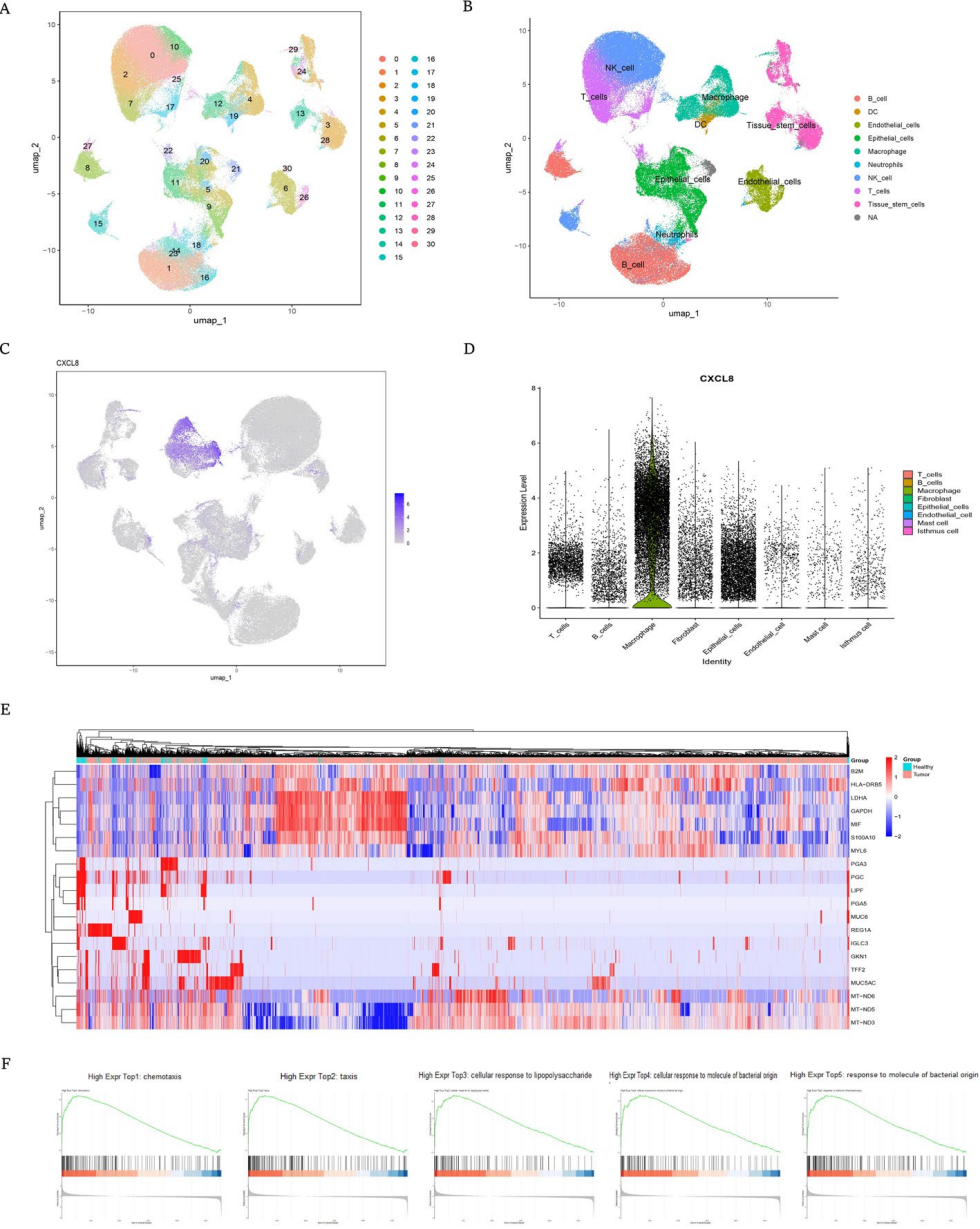
Single-Cell Analysis Reveals Macrophages as the Primary Source of *CXCL8* and Delineates Its Functional Impact. **A** UMAP visualization showing the distribution of all cell clusters identified from single-cell RNA-sequencing data. **B** UMAP plot showing the annotation of major cell types, including B cells, dendritic cells, endothelial cells, epithelial cells, macrophages, neutrophils, NK cells, T cells, tissue stem cells, and unassigned cells. **C** Feature plot showing the expression distribution of *CXCL8* across the single-cell landscape. **D** Violin plot showing *CXCL8* expression levels across different cell populations, indicating that macrophages exhibit the highest expression. **E** Heatmap showing the expression patterns of representative CXCL8-associated expressed genes across samples grouped as Healthy and Tumor. **F** Gene set enrichment analysis (GSEA) showing the principal enriched biological processes associated with differential CXCL8-related transcriptional activity

To characterize cellular heterogeneity and identify the source of the model’s chemokines, scRNA-seq was performed. Unbiased clustering and cell-type annotation revealed the major cell populations within the tumor microenvironment, including T cells, B cells, macrophages, endothelial cells, fibroblasts, and epithelial cells (Fig. 5A, B).

Mapping the expression of key genes from the prognostic model onto the single-cell atlas revealed that *CXCL8*, a central component of the risk signature, was predominantly and specifically expressed in macrophages (Fig. 5C, D). To assess functional heterogeneity, macrophages were subdivided into CXCL8-high and *CXCL8*-low groups. Differential expression analysis between these subsets identified distinct sets of regulated genes (Fig. 5E).

GSEA further demonstrated that *CXCL8*-high macrophages were significantly enriched in pathways associated with inflammation, immune responses, and oncogenic signaling (Fig. 5F, G).

### Pseudotime and cell communication analysis highlights macrophage function

**Fig. 6:**
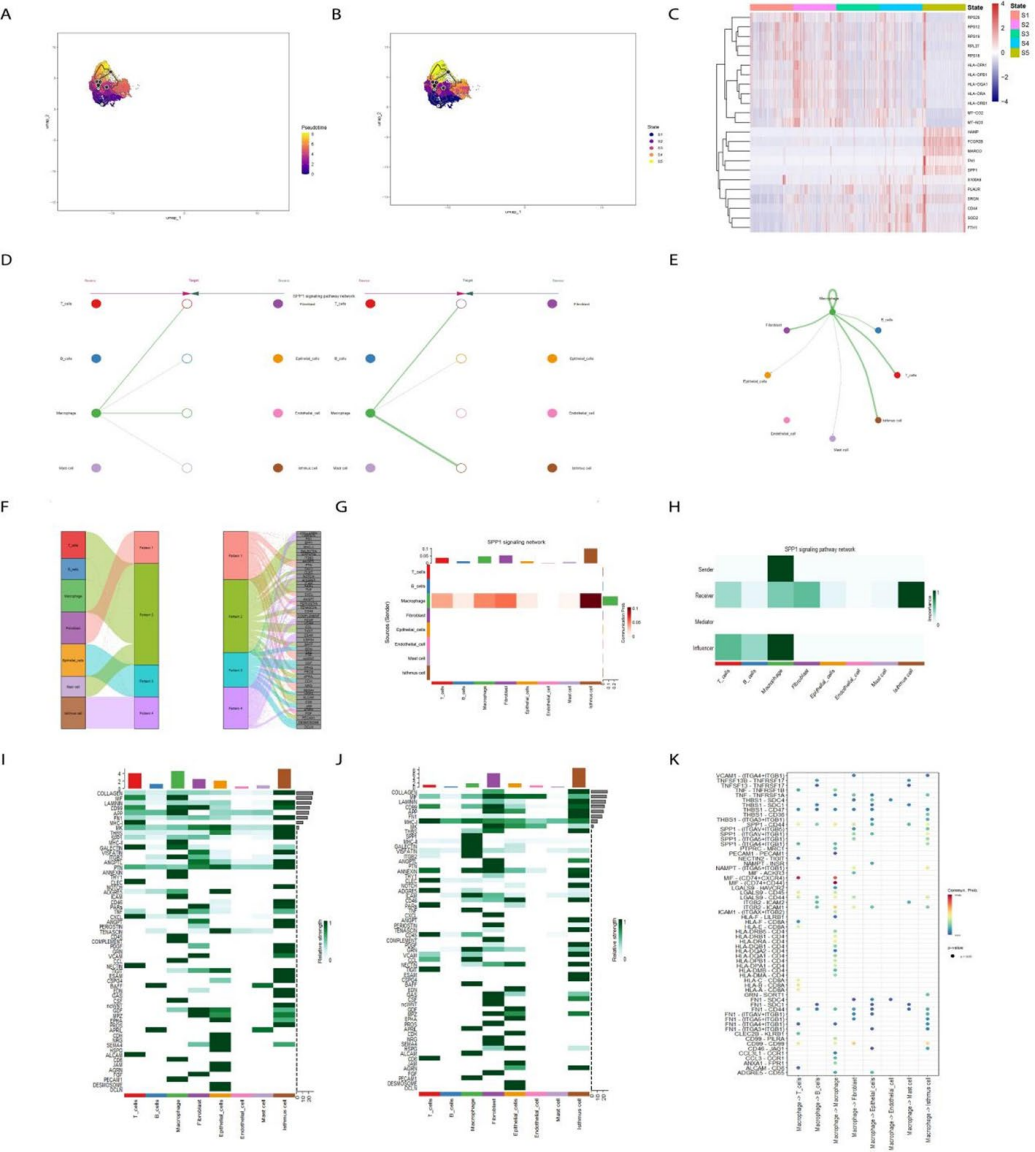
Pseudotime Analysis and Intercellular Communication of Macrophages. **A** Developmental trajectory of macrophages constructed using Monocle3, revealing potential differentiation or activation paths. **B** Pseudotime trajectory plot colored by macrophage sub-clusters, showing the distribution of different subtypes during state transition. **C** Heatmap of differentially expressed genes between pseudotime states (State 1 vs. State 3), showing changes in functional gene expression during macrophage transition. **D**, **E** Overview of the intercellular communication network analyzed by CellChat, illustrating the strength and direction of signaling among immune cell populations. **F** Visualization of the four major outgoing and incoming signaling patterns in the cellular communication network. **G**, **H** Detailed network diagrams of the SPP1 signaling pathway, highlighting its role in communication. **I** Heatmap displaying the differential strength of incoming signaling interactions for each cell type. **J** Heatmap displaying the differential strength of outgoing signaling interactions for each cell type. **K** Circle plot showing the probability of ligand-receptor-mediated communication between macrophage subtypes and other immune cell populations

To investigate the dynamic states of *CXCL8*-expressing macrophages, pseudotime trajectory analysis was performed. The results revealed a potential differentiation or activation continuum, suggesting continuous state transitions within the macrophage population (Fig. 6A). Mapping macrophage subclusters along this trajectory allowed visualization of their distribution over pseudotime (Fig. 6B). Gene expression analysis along the trajectory identified dynamically regulated genes, with one gene set associated with early states (State 1) and another with late states (State 3), indicating functional reprogramming during macrophage progression (Fig. 6C).

Intercellular communication was further examined using CellChat. The global network analysis revealed extensive signaling interactions among macrophages, T cells, and endothelial cells (Fig. 6D, E). Four major outgoing and incoming signaling patterns were identified, highlighting macrophages as central signaling hubs within the TME (Fig. 6F). Notably, the SPP1 signaling pathway emerged as a key communication axis originating from macrophages (Fig. 6G, H and Figure S5). Differential signaling strength analysis demonstrated that macrophages exhibited significantly higher incoming and outgoing interactions compared with other cell populations, particularly in ligand–receptor-mediated communication with T cells and endothelial cells (Fig. 6I–K).

### Supportive validation for the model

Functional enrichment analysis of DEGs between high- and low-risk groups revealed significant overrepresentation of GO terms and KEGG pathways associated with the extracellular matrix, cell adhesion, and cancer-related signaling pathways in the high-risk group (Supplementary Figure S1). Bulk immune infiltration analysis using CIBERSORT demonstrated differential proportions of 22 immune cell types between risk groups, while drug sensitivity analysis indicated reduced responsiveness to specific chemotherapeutic agents in the high-risk cohort (Figure S2).

The prognostic relevance of the individual model component, *CXCL8*, was validated in an independent GEO cohort (GSE84437). Low *CXCL8* expression was associated with poorer prognosis and remained an independent prognostic factor in multivariate analysis (Figure S3). Further investigation of *CXCL8*-correlated genes in bulk transcriptomic data highlighted enrichment in pathways related to cell migration and immune regulation (Figure S4).

Immunohistochemical analysis of gastric cancer clinical samples demonstrated variable *CXCL8* expression across tumor subtypes, with low expression in mixed and intestinal-type tumors and elevated expression in diffuse-type tumors (Figure S6).

To corroborate computational predictions, independent in vitro experiments were performed. Transwell assays revealed that macrophage co-culture significantly enhanced GC cell migration and invasion, effects that were partially reversed by *CXCL8* neutralization (Figure S7). PMA-differentiated THP-1 cells exhibited higher *CXCL8* mRNA expression and protein secretion compared with MKN45 cells (Figure S8A, B), and co-culture increased tumor cell viability in a *CXCL8*-dependent manner (Figure S8C). Moreover, macrophage co-culture induced *PD-L1* upregulation in GC cells, which was attenuated upon *CXCL8* blockade (Figure S9), supporting a macrophage-derived *CXCL8*–mediated pro-tumorigenic phenotype.

Drug sensitivity assays further demonstrated that macrophage co-culture reduced GC cell sensitivity to L-OHP and 5-FU, as indicated by shifts in dose–response curves and increased IC₅₀ values (Figure S10). Notably, *CXCL8* neutralization partially restored chemosensitivity, highlighting the functional role of macrophage-derived *CXCL8*8 in modulating chemotherapy response.

## Discussion

Chemokines and their receptors have emerged as promising biomarkers for gastric cancer prognosis [21]. In this study, we analyzed the relationship between chemokine expression patterns and gastric cancer outcomes and developed a chemokine-based prognostic model for patient stratification. The model was further evaluated for its association with the TME and potential clinical applicability. Our findings indicate that chemokines play pivotal roles in gastric cancer progression and immune evasion, with *CXCL8* expression strongly correlating with patient prognosis.

By integrating TCGA and transcriptomic datasets, six key prognostic chemokines—including *CXCL1*, *CXCL8*, and *CCL21*—were identified, forming the basis of a risk score validated as an independent predictor of clinical outcome. This risk score was significantly associated with clinicopathological characteristics. Moreover, a comprehensive nomogram incorporating TNM stage, risk score, age, and pathological classification was constructed, providing a practical and innovative tool for individualized prognosis prediction in gastric cancer patients.

To elucidate the mechanisms underlying prognostic differences, pathway enrichment analyses were conducted. High-risk patients showed significant enrichment of neuroactive ligand–receptor interaction pathways, suggesting dysregulated neuroendocrine signaling in gastric cancer progression [22]. They also exhibited enrichment in cell adhesion molecule pathways, reflecting disrupted intercellular adhesion. Abnormal expression of adhesion molecules can weaken cell–cell contacts, facilitating tumor cell detachment from the primary site and promoting invasion and metastasis [23, 24]. Furthermore, the PI3K–Akt signaling pathway—commonly hyperactivated in gastric cancer—was enriched in high-risk patients, indicating its role in tumor progression and therapeutic resistance and highlighting its potential as a therapeutic target [25–28].

A recent study indicated that chemokines do not directly regulate mutation accumulation but instead shape an immune environment that recognizes and targets tumor neoantigens generated by a high mutational load during immunotherapy [29]. Analyses of TCGA and ICGC gastric cancer cohorts have shown that frequently mutated genes, such as *FAT4*, are significantly associated with TMB and clinical outcomes, supporting the prognostic relevance of TMB in gastric cancer [30]. Recent studies have demonstrated that anticancer bioactive peptides can induce apoptosis in gastric cancer cells through activation of the *TP53* signaling cascade, highlighting *TP53* as a critical regulator of tumor cell fate [31]. Within the high-risk cohort, *TTN* and *TP53* exhibited elevated mutation frequencies (45%), suggesting that these patients may benefit from anti-PD-1/PD-L1 immunotherapy [32].

Comprehensive characterization of the tumor microenvironment remains essential for identifying novel therapeutic strategies in gastric cancer [33, 34]. Using the CIBERSORT algorithm, we observed distinct immune cell infiltration patterns between risk groups. Low-risk patients exhibited increased CD8⁺ T cells, activated dendritic cells, and eosinophils, which may enhance immune surveillance and tumor clearance. In contrast, high-risk individuals showed elevated levels of T cells, monocytes, and neutrophils. Pharmacological sensitivity analysis revealed higher IC50 values for standard chemotherapeutic agents—including 5-fluorouracil, Afatinib, Acetalax, and Bortezomib—in the high-risk group, potentially contributing to increased therapeutic resistance [35].

Validation using the GSE84437 dataset highlighted a significant association between *CXCL8* expression and clinical parameters, including age, gender, and tumor stage. Reduced *CXCL8* expression was linked to poorer prognosis. Analysis of *CXCL8*-related genes revealed marked upregulation of MMP7, which promotes tumor invasion and metastasis via extracellular matrix degradation and disruption of E-cadherin, weakening intercellular junctions and facilitating tumor dissemination [36]. Upregulation of GABRP was also observed, enhancing tumor proliferation and migration through PI3K–Akt signaling [37]. In contrast, *CDX1* and *CDX2*, key regulators of intestinal epithelial differentiation, were downregulated, correlating with aggressive gastric cancer progression [38]. Additionally, downregulation of CDH17, a gene involved in cell–cell adhesion, may impair cellular adhesion and promote tumor cell migration [39].

Emerging evidence across multiple cancer types indicates that reduced expression of chemokines or their receptors is consistently associated with impaired immune cell recruitment and poor clinical outcomes [40]. In pancreatic and bladder cancers, diminished CXC chemokine or CXCR6 signaling reflects inadequate immune orchestration, characterized by reduced infiltration of cytotoxic T cells, NK cells, and antigen-presenting cells [40, 41]. Although CXCL8 is well recognized for its pro-angiogenic role across various malignancies [42, 43], our findings demonstrate that decreased *CXCL8* expression is significantly associated with higher mortality in gastric cancer. This paradox may reflect CXCL8’s complex, dose-dependent effects during tumor progression: in early stages, CXCL8 promotes anti-tumor immunity by recruiting macrophages and T lymphocytes [44], whereas sustained overexpression can drive chronic inflammation and activate pro-tumor pathways such as NF-κB [45].

Our single-cell analysis provides crucial insight into this phenomenon, identifying macrophages as the predominant source of *CXCL8* within the gastric cancer tumor microenvironment. This suggests a potential autocrine or paracrine feedback loop, in which macrophage-derived *CXCL8* shapes the immune landscape. Furthermore, *CXCL8*-high macrophages displayed a distinct pro-inflammatory and pro-angiogenic phenotype, marked by significant upregulation of IL1B, CCL2, CXCL2, and the angiogenic factor VEGFA. These observations support the dual role of *CXCL8*, highlighting that its context-dependent function is tightly linked to a specific macrophage subpopulation that simultaneously promotes inflammation and angiogenesis. When *CXCL8* expression falls below critical thresholds in advanced disease, immune evasion may ensue, compromising immune surveillance. Tumors may compensate via upregulation of alternative chemokines, such as CXCL1 and CXCL3, which facilitate immunosuppressive M2 macrophage infiltration [46–49].

From a public health perspective, gastric cancer poses a substantial global health burden due to its high incidence and poor prognosis [50]. Despite advances in screening and diagnostic technologies, most patients are diagnosed at advanced stages, contributing to persistently low five-year survival rates [51]. Therefore, the identification of novel biomarkers and development of robust prognostic models remain critical for improving early detection and guiding personalized treatment strategies. Our chemokine-based prognostic model provides enhanced predictive capability for patient outcomes, supporting clinical decision-making. In particular, *CXCL8*, as an independent prognostic factor, may enable clinicians to identify high-risk individuals and tailor individualized therapeutic interventions.

In therapeutic contexts, modulation of chemokine expression represents a promising immunotherapeutic strategy for gastric cancer [52]. With the increasing use of immune checkpoint inhibitors [53], evidence suggests that *CXCL8* is closely associated with mechanisms of immune evasion, potentially influencing immunotherapy efficacy by regulating immune cell infiltration and function within the tumor microenvironment. Future studies should investigate the relationship between *CXCL8* expression and immunotherapy response, evaluating its potential as a therapeutic target. Furthermore, our findings indicate that *CXCL8* may be closely linked to the infiltration patterns of multiple immune cell types, including CD8⁺ T cells and dendritic cells, offering novel insights for precise modulation of the tumor immune microenvironment.

Our in-depth investigation of the tumor microenvironment using single-cell analysis further clarified the complex cellular dynamics and intercellular communication networks [34]. Pseudotime analysis delineated the developmental trajectories of macrophages, revealing potential differentiation and activation pathways within the tumor and highlighting the dynamic functional states of macrophages during gastric cancer progression. Importantly, intercellular communication analysis via CellChat identified macrophages as central hubs within the TME signaling network. The SPP1 signaling pathway emerged as a key communication axis, with *CXCL8*-expressing cells—primarily macrophages—signaling to other cell types, while macrophages themselves were the main signal recipients. This suggests a feedback mechanism whereby macrophages regulate both their own function and the broader immune response through SPP1 signaling. Additionally, interactions such as MIF-(CD74⁺CXCR4) and MIF-(CD74⁺CD44) further emphasize macrophages as master regulators of intercellular communication. Collectively, these findings provide a detailed mechanistic view of how *CXCL8*-associated macrophage populations may shape the distinct immune landscapes observed between high- and low-risk patients, highlighting potential targets for disrupting pro-tumorigenic networks.

Despite significant advances, this study has several limitations. First, analyses relied on retrospective data from public repositories, which may introduce selection biases affecting result accuracy. Second, the precise mechanisms underlying *CXCL8*’s role in the gastric cancer immune microenvironment remain incompletely understood, highlighting the need for future studies using animal models and clinical specimens to validate its functions in immune evasion, tolerance, and suppression. Third, the relationships between *CXCL8* and other immune checkpoint molecules, such as PD-1 and CTLA-4, require further investigation, particularly regarding potential interactions with immune escape mechanisms that may influence therapeutic outcomes. Finally, although our study primarily utilized transcriptomic data, future multi-omics approaches could offer deeper insights into *CXCL8*-related pathways and its complex roles within the tumor microenvironment, potentially guiding the development of novel targeted therapies.

## Conclusion

In summary, this study systematically evaluated the role of chemokines in gastric cancer and developed a novel prognostic scoring model, which demonstrated clinical applicability. Notably, *CXCL8* was identified as a key chemokine, with low expression closely associated with poor patient prognosis. This effect may be mediated through modulation of the tumor immune microenvironment and facilitation of tumor metastasis. Future studies are warranted to elucidate the underlying mechanisms of *CXCL8* and to assess its potential as a target in immunotherapy, offering new insights for personalized therapeutic strategies in gastric cancer.

## Supplementary Information

Below is the link to the electronic supplementary material.

Supplementary Material 1.

## Data Availability

This study analyzed publicly available datasets. The gastric cancer cohort was obtained from The Cancer Genome Atlas (TCGA) Stomach Adenocarcinoma project (TCGA-STAD), available at: https://portal.gdc.cancer.gov/projects/TCGA-STAD. Two additional transcriptomic datasets, GSE183904 and GSE84473, were downloaded from the Gene Expression Omnibus (GEO) database at: https://www.ncbi.nlm.nih.gov/geo. Databases and Gene Sets Used for Analysis: The following publicly available resources were used for specialized analyses: CIBERSORT LM22 Signature Matrix (for immune infiltration analysis): https://cibersort.stanford.edu/. Molecular Signatures Database (MSigDB) (for GSEA and ssGSEA gene sets, including “c2.cp.kegg.v2023.1.Hs.symbols.gmt” and “h.all.v2023.1.Hs.symbols.gmt”): https://www.gsea-msigdb.org/gsea/msigdb/. Kyoto Encyclopedia of Genes and Genomes (KEGG) Gene Sets (for GO and KEGG enrichment analysis): https://www.genome.jp/kegg/. Genomics of Drug Sensitivity in Cancer (GDSC) Database (release 2023, for drug sensitivity analysis): https://www.cancerrxgene.org/.

## Abbreviations

ANOVA: Analysis of variance
DCA: Decision curve analysis
DEGs: Differentially expressed genes
DEPCs: Differentially expressed prognostic chemokine factors
FDR: False discovery rate
GC: Gastric cancer
GDSC: Genomics of drug sensitivity in cancer
GEO: Gene Expression Omnibus
GO: Gene Ontology
GSEA: Gene set enrichment analysis
GSVA: Gene set variation analysis
IC50: Half maximal inhibitory concentration
KEGG: Kyoto Encyclopedia of Genes and Genomes
NES: Normalized enrichment score
OS: Overall survival
PCA: Principal component analysis
PFS: Progression-free survival
ROC: Receiver operating characteristic
scRNA-seq: Single-cell RNA sequencing
SNN: Shared nearest neighbor
ssGSEA: Single-sample gene set enrichment analysis
TCGA: The Cancer Genome Atlas
TMB: Tumor mutation burden
TME: Tumor microenvironment
UMAP: Uniform Manifold Approximation and Projection
