# Supplementary material for: CXCL8 producing macrophages shape gastric cancer outcomes
Source: Discov Oncol. 2026 Jun 17;17:1077. doi: 10.1007/s12672-026-05452-9 (PMC13400654; doi:10.1007/s12672-026-05452-9)

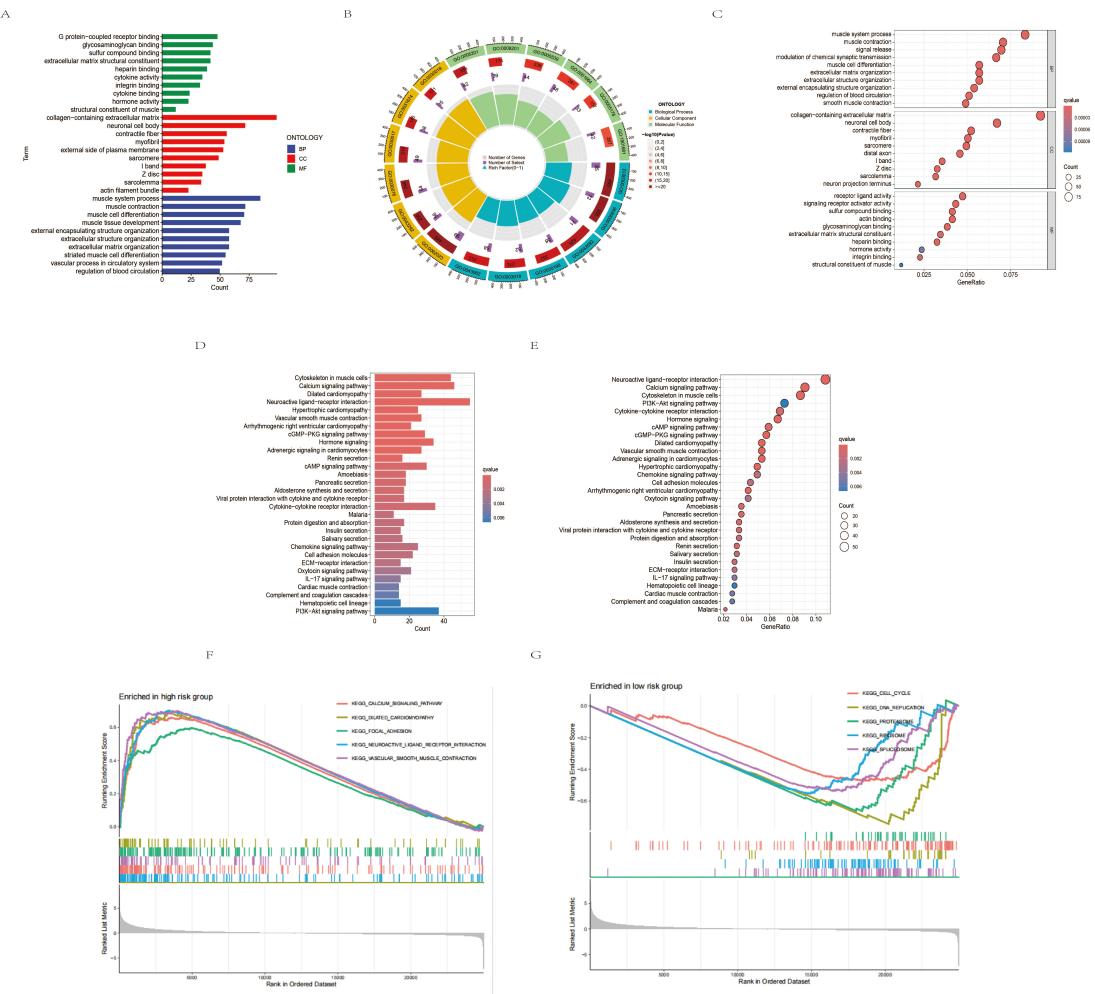


Figure S1: Potential mechanism analysis of chemokine related gene features.

(A) GO Function Bar Chart

(B) GO Circle Diagram

(C) GO Enrichment Bubble Chart

(D) KEGG Bar Chart

(E) KEGG Bubble Chart

(F) GSEA pathway enrichment map for high-risk groups

(G) Enrichment map of GSEA pathway in low-risk group.


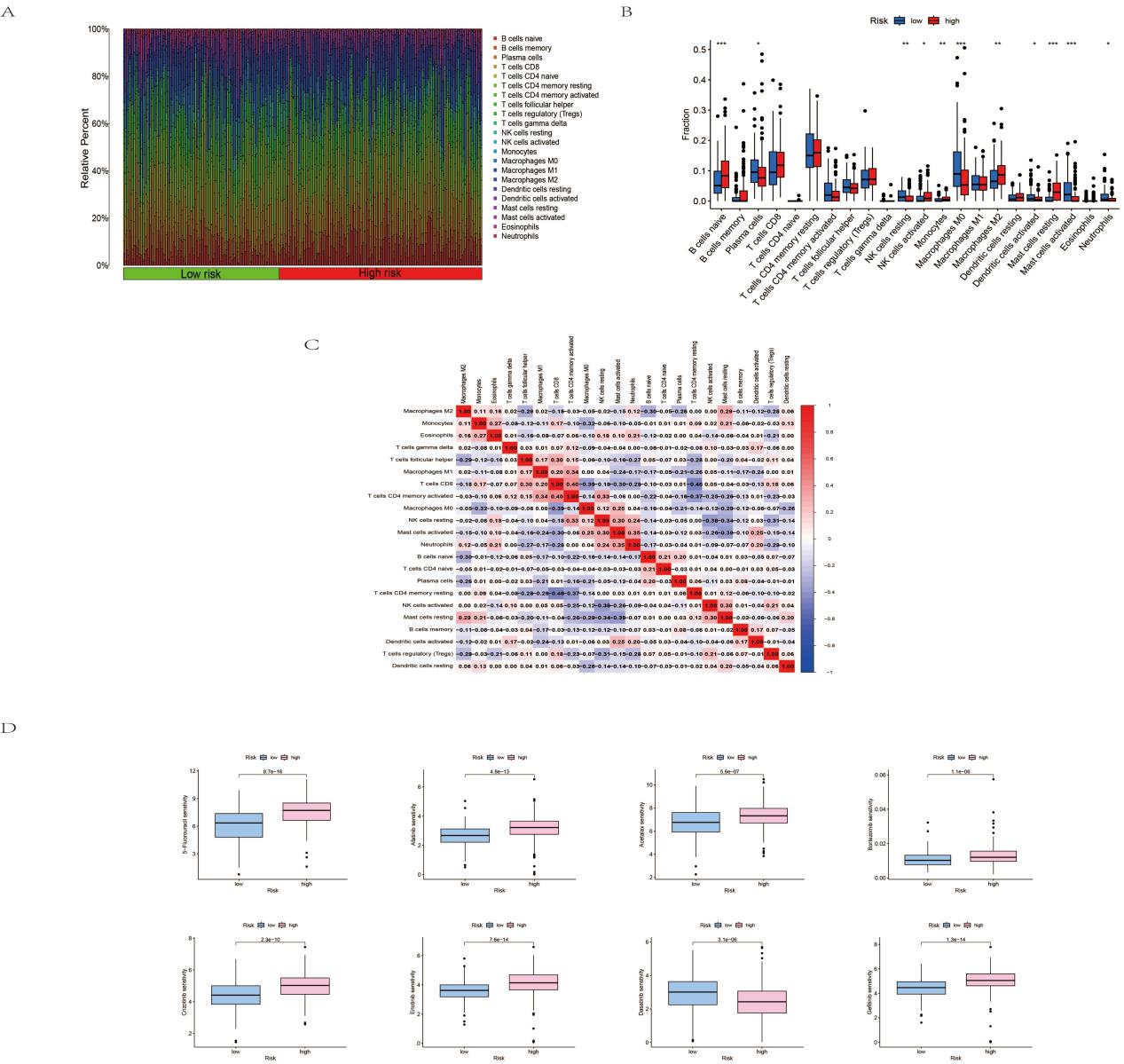


Figure S2: Characteristics of immune cell infiltration and drug sensitivity analysis in chemokine related subgroups.

(A) The expression ratio of 22 immune cells in low-risk and high-risk groups.

(B) The box plot illustrates the differential expression of immune checkpoint genes in high-risk and low-risk categories.

(C) Correlation diagram of immune cell expression.

(D) Differential analysis of drug sensitivity between low-risk and high-risk groups (5-Fluorouracil, Afatinib, Acetaax, Bortezomib, Crizotinib, Erlotinib, Dasatinib, Gefitinib)


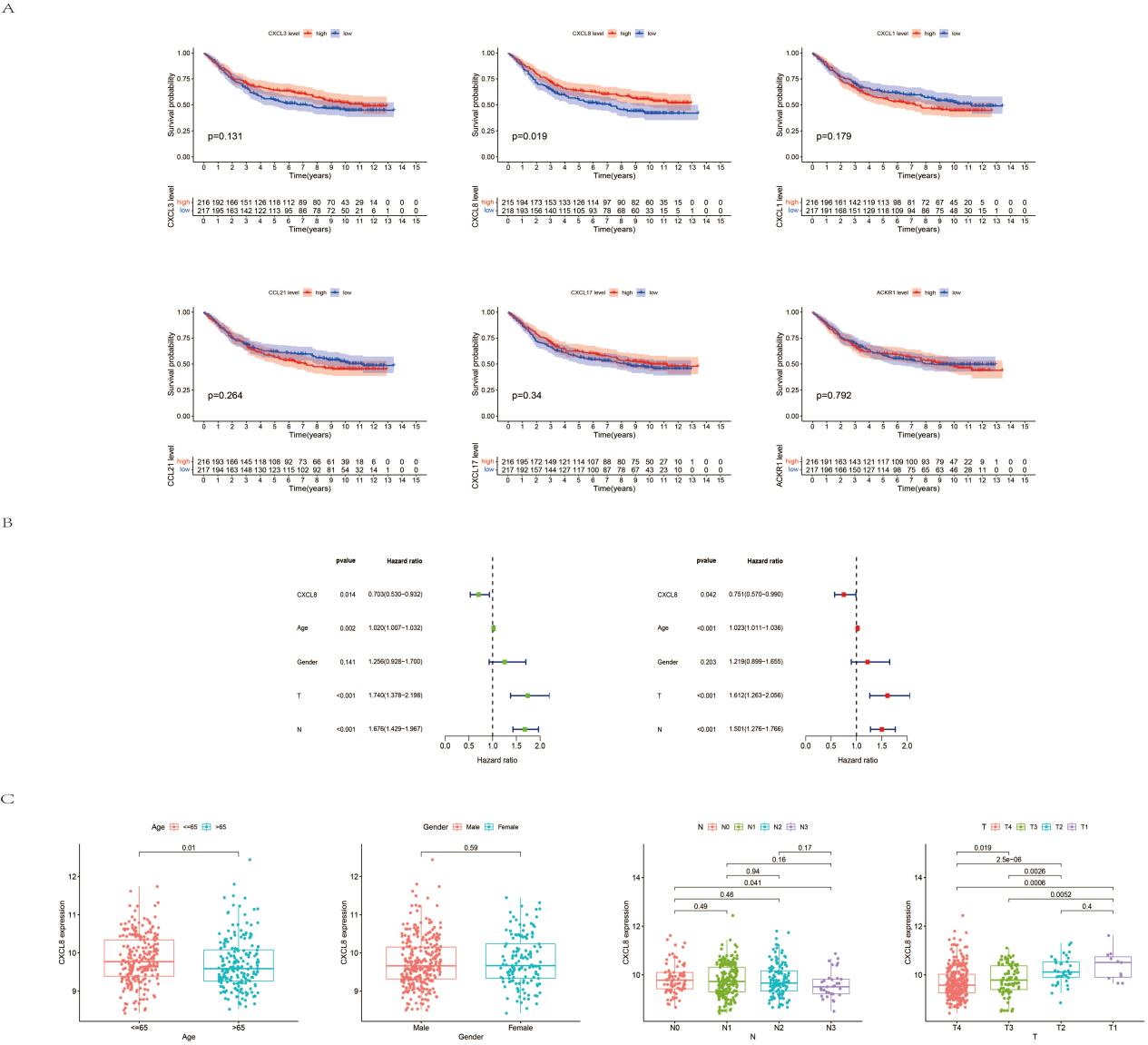


Figure S3: Validation of the chemokine related prognostic model.

(A) The Kaplan Meier survival plot depicts the OS in the GSE84437 cohort.

(B) The results of univariate and multivariate Cox regression evaluation of *CXCL8* expression levels and clinical pathological attributes in the cohort.

(C) Clinical correlation analysis of *CXCL8* expression level in the cohort.


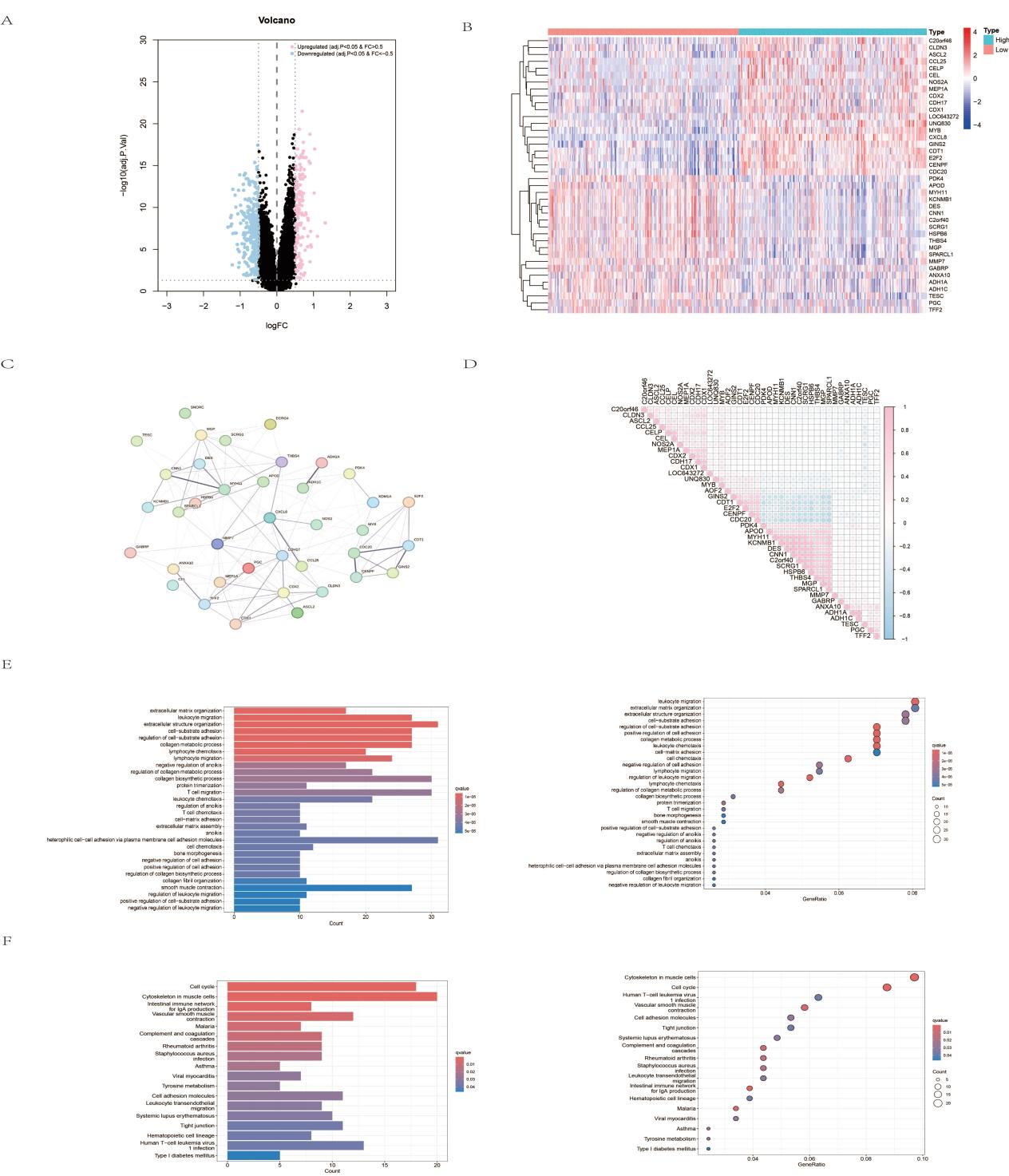


Figure S4: Identification and pathway enrichment of *CXCL8* related genes.

(A) Volcano plot of significantly related genes between high *CXCL8* expression group and low *CXCL8* expression group.

(B) A heatmap of 20 genes significantly upregulated and 20 genes significantly downregulated in the high *CXCL8* expression group and low *CXCL8* expression group.

(C) A PPI network was constructed by plotting 20 genes that were significantly upregulated and 20 genes that were significantly downregulated in the high *CXCL8* expression group and low *CXCL8* expression group.

(D) Correlation analysis of 20 genes significantly upregulated and 20 genes significantly downregulated in the high *CXCL8* expression group and low *CXCL8* expression group.

(E) GO enrichment analysis of 20 genes significantly upregulated and 20 genes significantly downregulated in the high *CXCL8* expression group and low *CXCL8* expression group.

(F) KEGG enrichment analysis of 20 genes significantly upregulated and 20 genes significantly downregulated in the high *CXCL8* expression group and low *CXCL8* expression group.


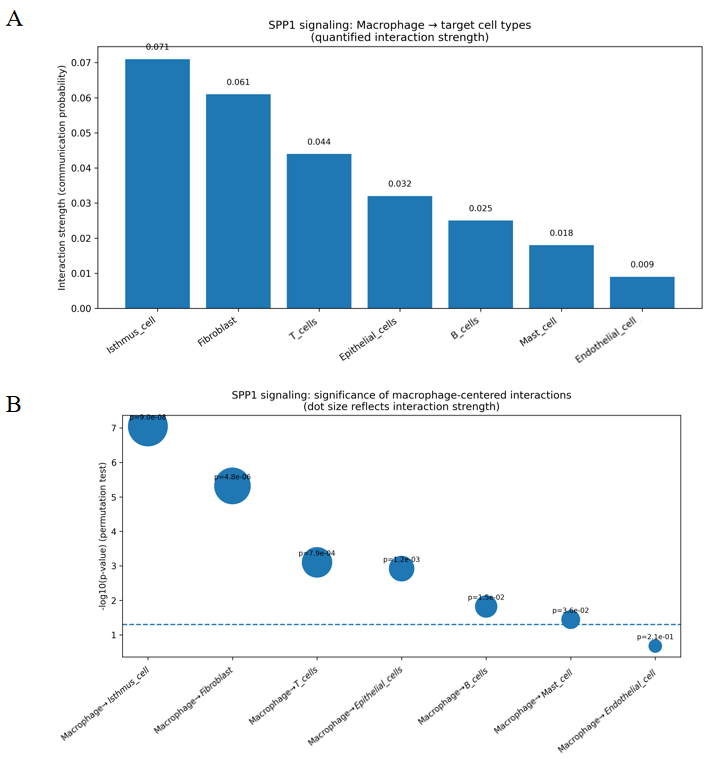


Figure S5: SPP1 signaling in macrophage-centered communication.

(A) Interaction strength values of SPP1-mediated cell–cell communication.

(B) Statistical significance of SPP1 signaling interactions.


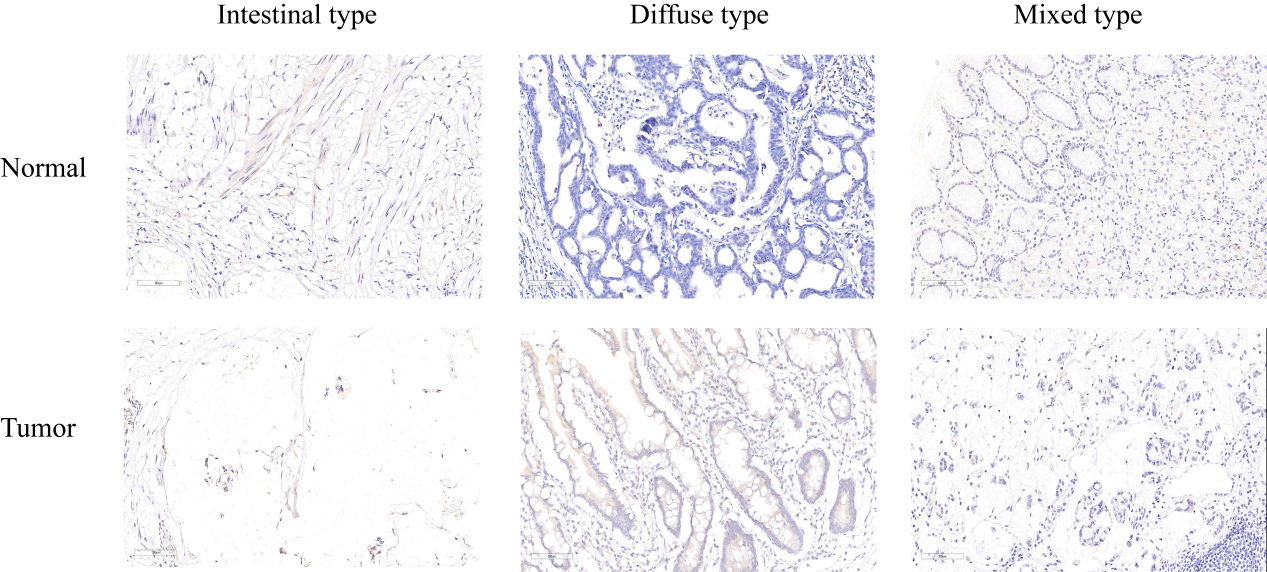


Figure S6: Immunohistochemical analysis of *CXCL8* expression in gastric cancer tissues. (×100 um)

Immunohistochemical staining in intestinal-type gastric cancer, diffuse-type gastric cancer, and adjacent normal tissues according to Lauren classification. Brown staining indicates positive expression of CXCL8. (scale bar = 100 μm).


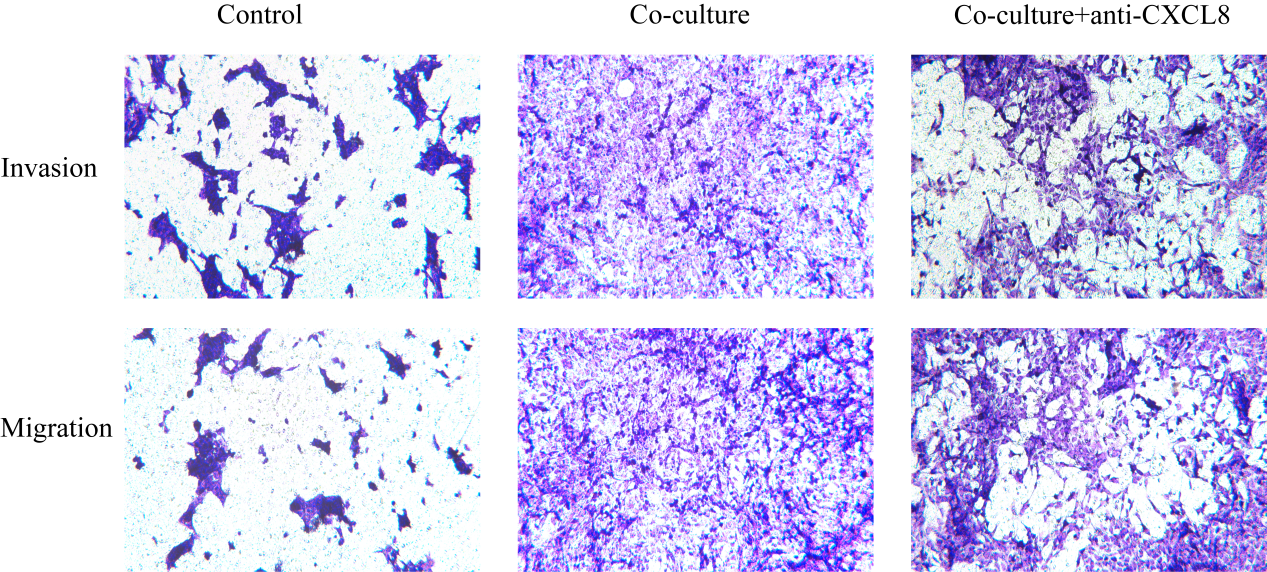


Figure S7: MKN45 cells, Transwell Invasion and Migration (×100 um)

Transwell migration and Matrigel invasion assays of MKN45 cells under control conditions, co-culture, and co-culture with anti-CXCL8 treatment. Migrated or invaded cells were stained with crystal violet.(scale bar = 100 μm).


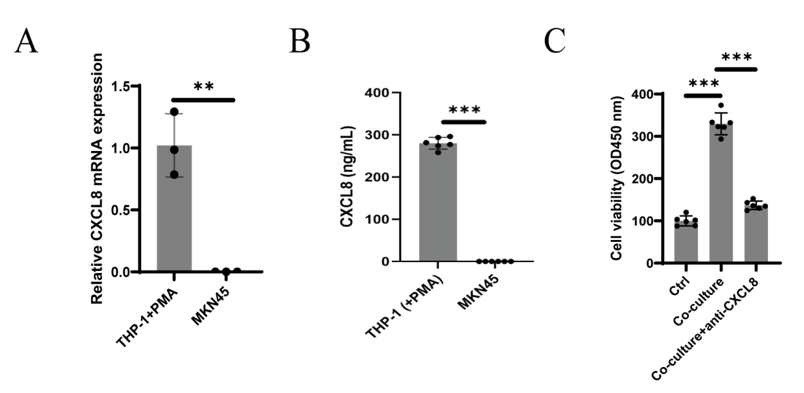


Figure S8: CXCL8 production and its functional effects in vitro.

(A) CXCL8 mRNA expression in PMA-differentiated THP-1 cells and MKN45 cells, as determined by quantitative PCR.

(B) CXCL8 protein levels in culture supernatants of PMA-differentiated THP-1 cells and MKN45 cells, measured by ELISA.

(C) Cell viability assessed by CCK-8 assay in control, co-culture, and co-culture plus anti-CXCL8 antibody conditions (OD 450 nm). **P < 0.01, ***P < 0.001.


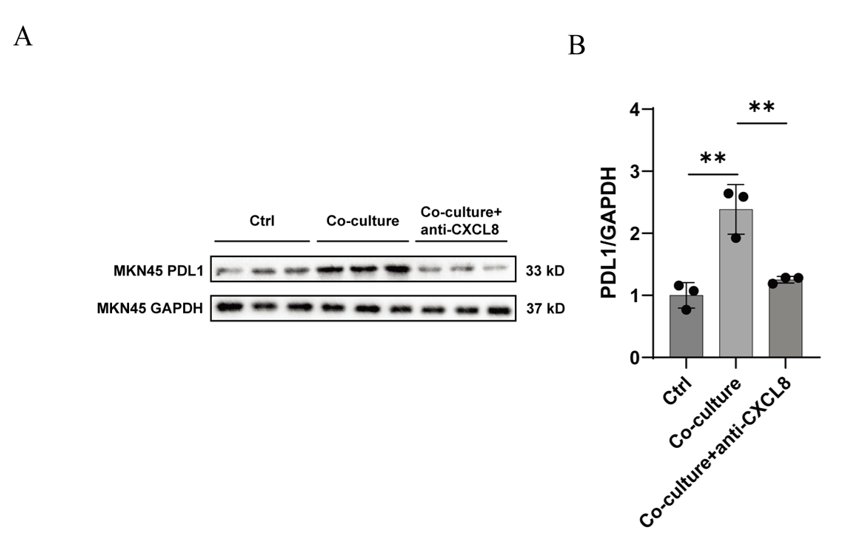


Figure S9: Western blot analysis of PD-L1 expression in MKN45 cells.

(A) Representative Western blot showing PD-L1 protein levels in MKN45 cells under control conditions (Ctrl), macrophage co-culture (Co-culture), and co-culture with CXCL8 neutralizing antibody (Co-culture + anti-CXCL8). GAPDH was used as the loading control. The molecular weights of PD-L1 (33 kDa) and GAPDH (37 kDa) are indicated on the right.

(B) Quantitative densitometric analysis of PD-L1 protein expression normalized to GAPDH. Data are presented as mean ± SD from independent experiments. Co-culture significantly increased PD-L1 expression compared with control, while CXCL8 neutralization partially reversed this effect. **P < 0.01.


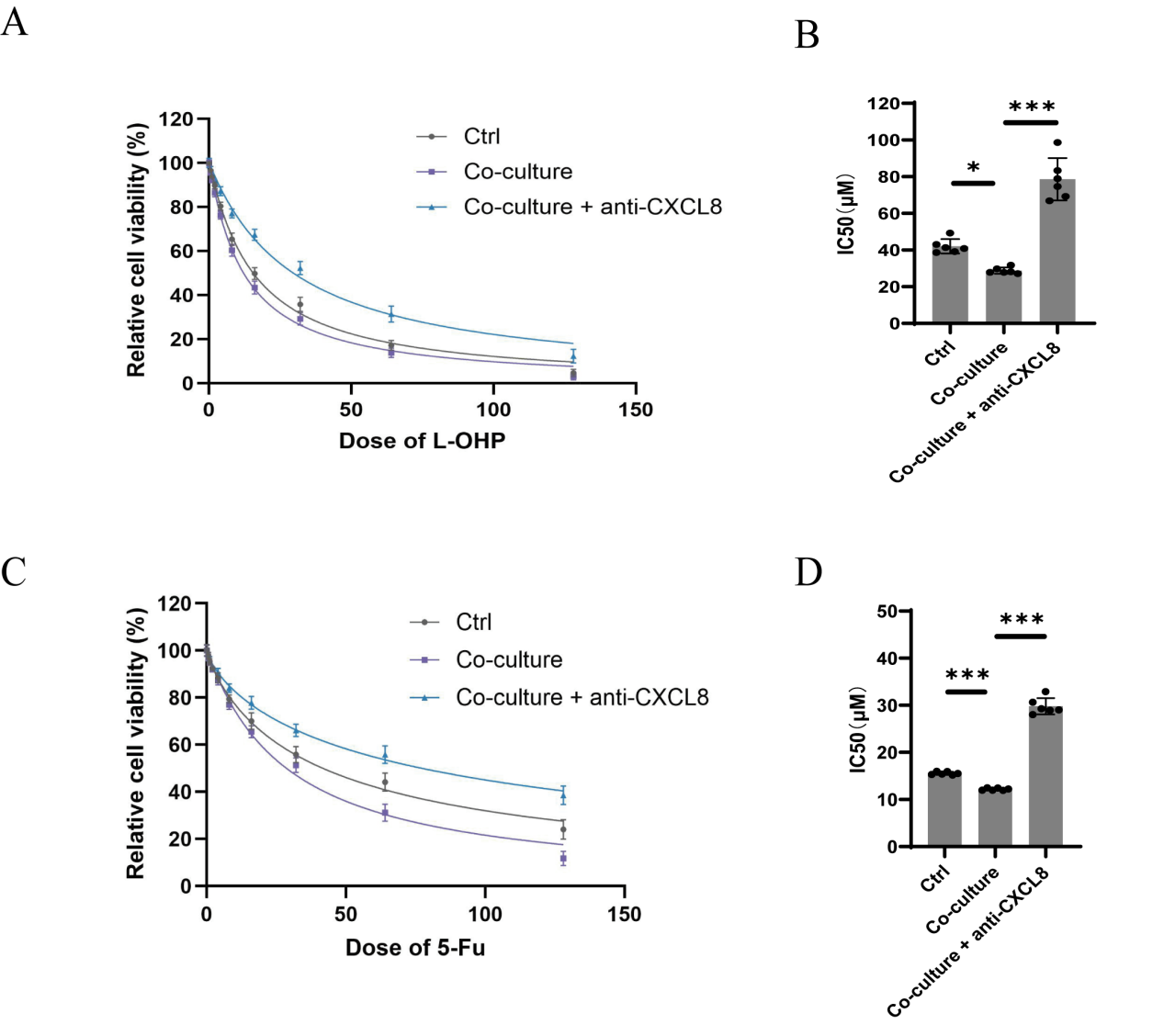


Figure S10: Chemotherapy sensitivity.

1. Dose–response curves of gastric cancer cells treated with L-OHP under control, co-culture, and co-culture plus anti-CXCL8 antibody conditions.
   (B) IC50 values of L-OHP calculated from the corresponding dose–response curves.
   (C) Dose–response curves of gastric cancer cells treated with 5-Fu under control, co-culture, and co-culture plus anti-CXCL8 antibody conditions.
   (D) IC50 values of 5-Fu calculated from the corresponding dose–response curves.
   Data are presented as mean ± SD. *P < 0.05, ***P < 0.001.

GADPH


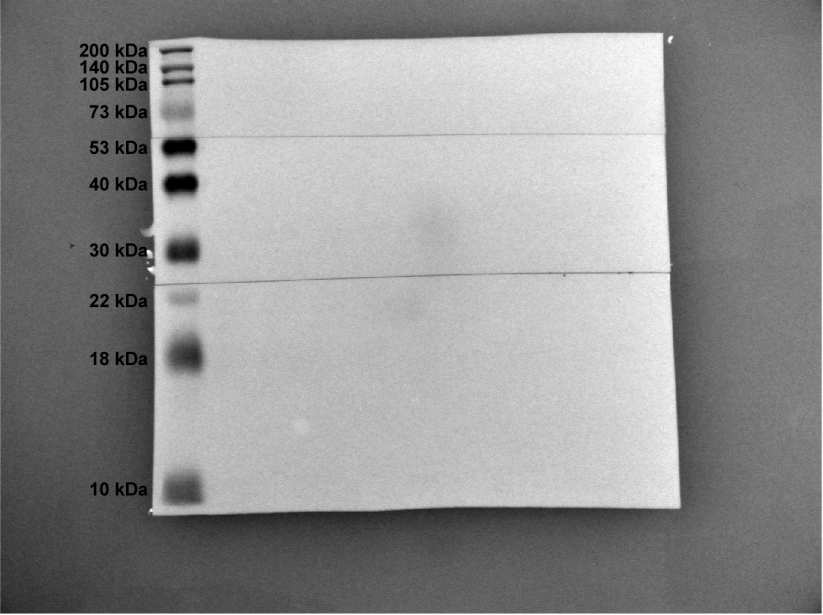

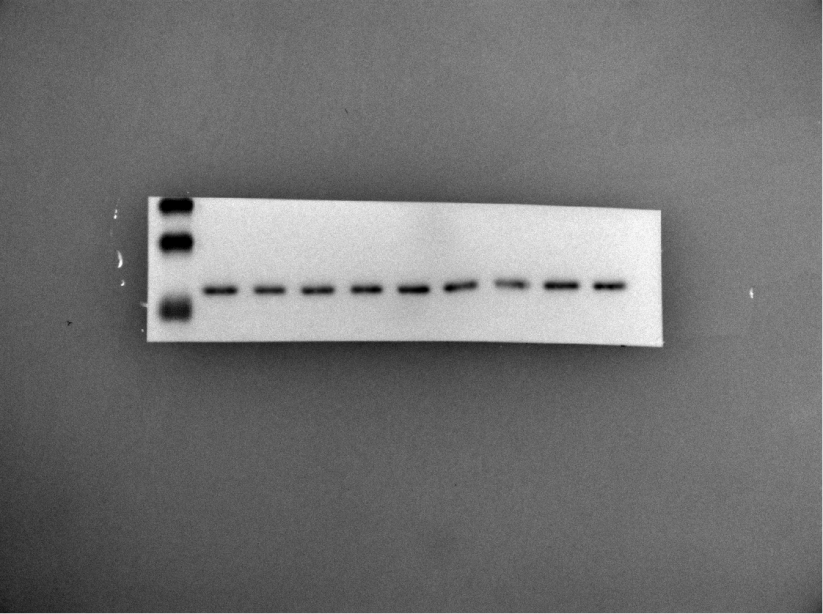


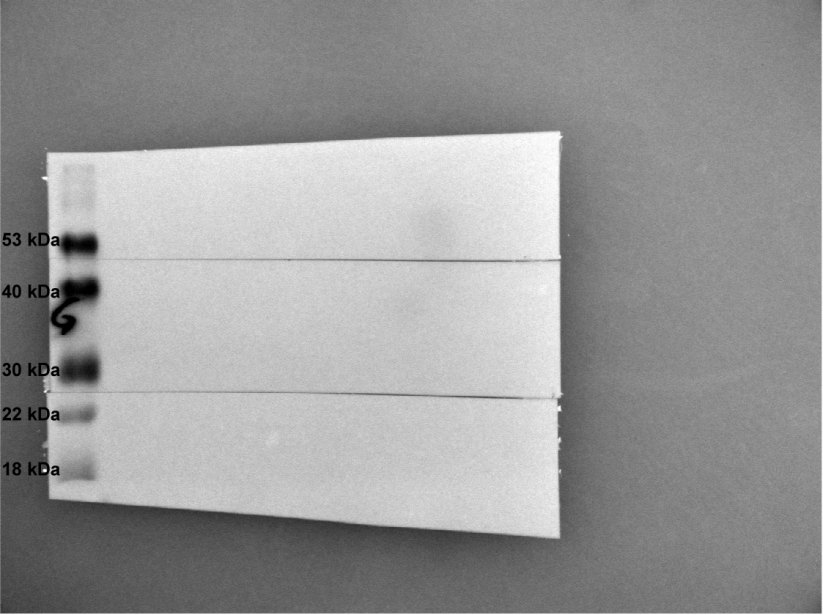

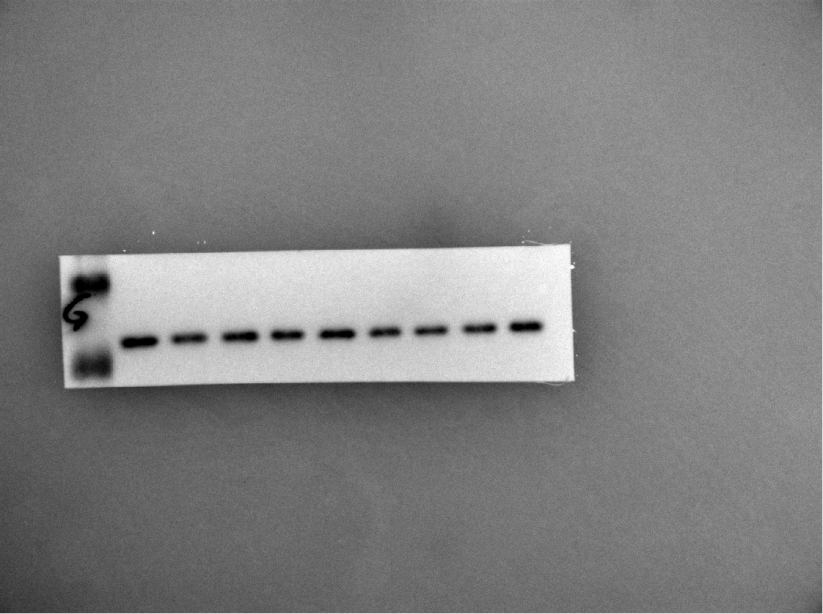


PDL1


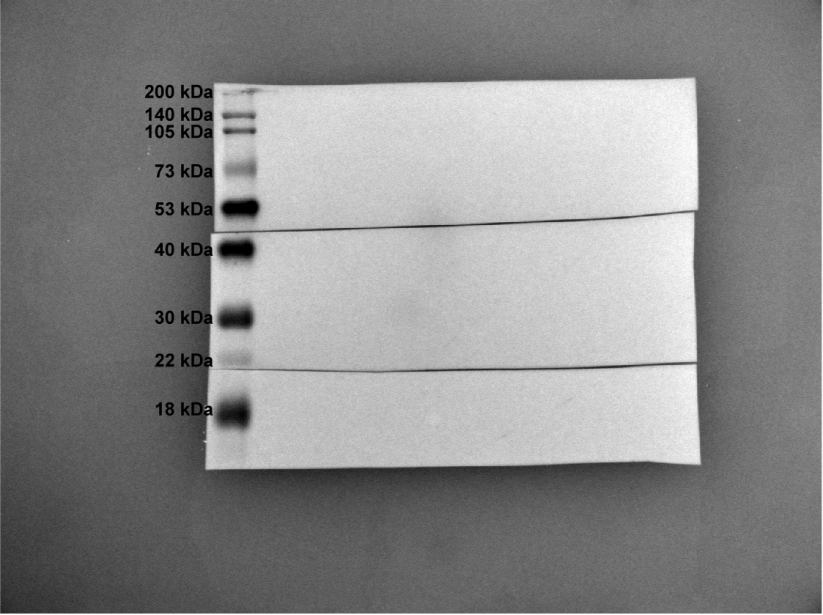

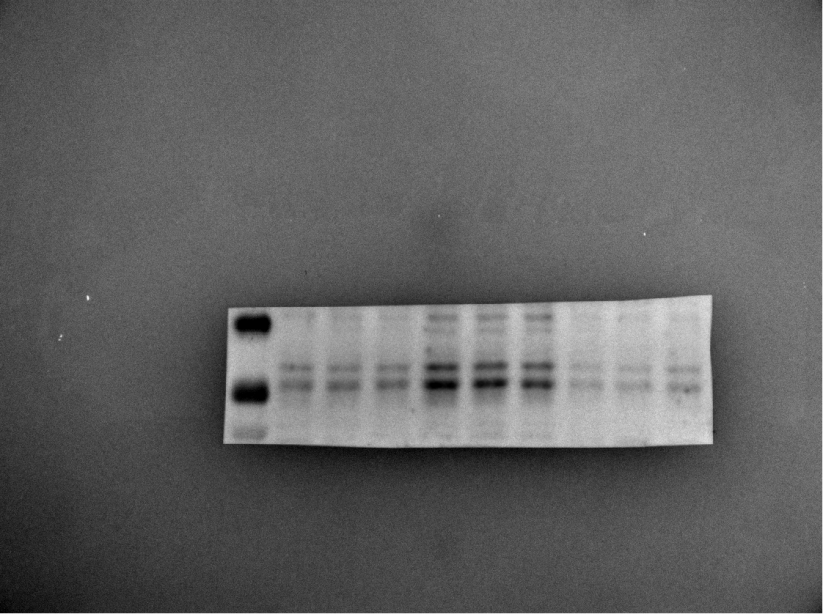


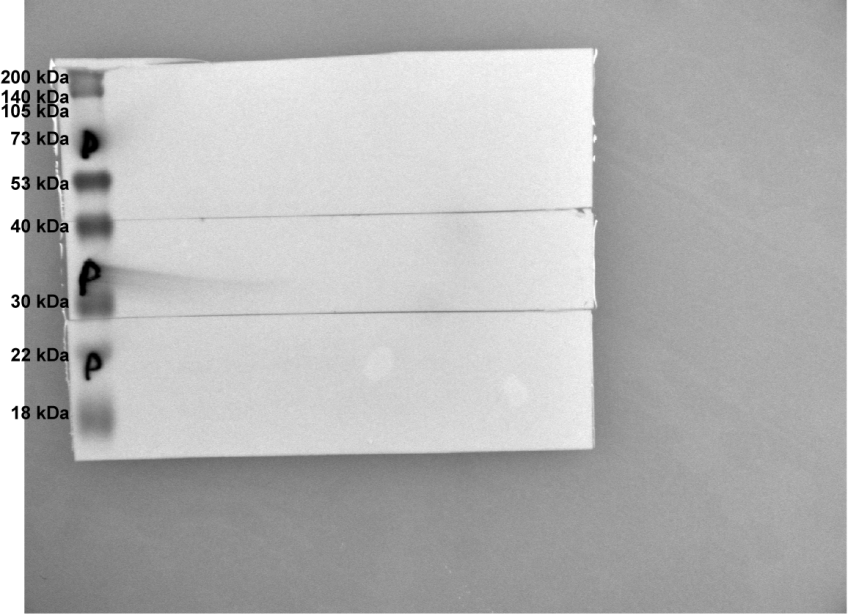

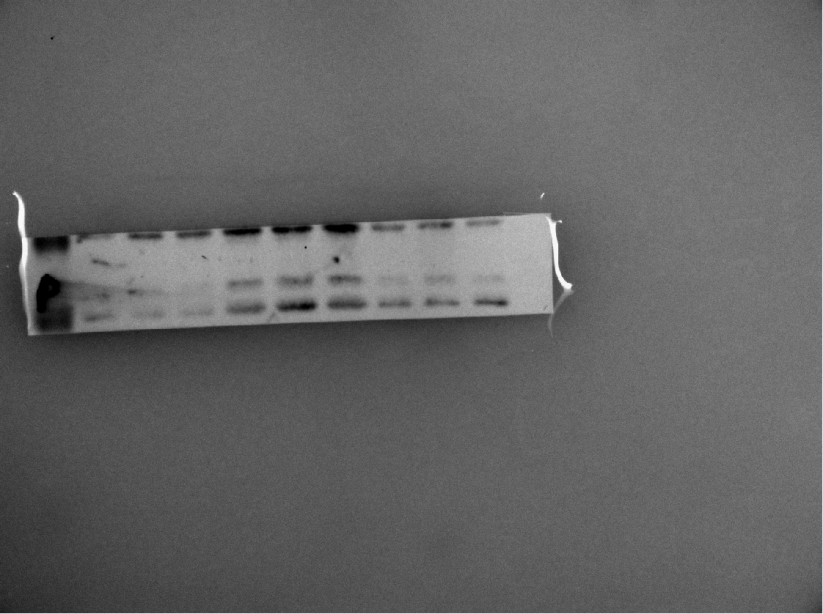

Supplement: Supplementary file 1 — Supplementary Material 1. [file 12672_2026_5452_MOESM1_ESM.docx]
